# Insights from a Box–Behnken Optimization Study of Microemulsions with Salicylic Acid for Acne Therapy

**DOI:** 10.3390/pharmaceutics14010174

**Published:** 2022-01-12

**Authors:** Maria-Cristina Anicescu, Cristina-Elena Dinu-Pîrvu, Marina-Theodora Talianu, Mihaela Violeta Ghica, Valentina Anuța, Răzvan-Mihai Prisada, Anca Cecilia Nicoară, Lăcrămioara Popa

**Affiliations:** 1 Department of Physical and Colloidal Chemistry, Faculty of Pharmacy, “Carol Davila” University of Medicine and Pharmacy, 020950 Bucharest, Romania; cristina.anicescu@drd.umfcd.ro (M.-C.A.); cristina.dinu@umfcd.ro (C.-E.D.-P.); marina-theodora.talianu@drd.umfcd.ro (M.-T.T.); valentina.anuta@umfcd.ro (V.A.); razvan.prisada@umfcd.ro (R.-M.P.); lacramioara.popa@umfcd.ro (L.P.); 2 Department of Pharmaceutical Technology and Biopharmacy, Faculty of Pharmacy, “Carol Davila” University of Medicine and Pharmacy, 020950 Bucharest, Romania; anca.nicoara@umfcd.ro

**Keywords:** O/W microemulsions, Box–Behnken design, rheological evaluation, DLS analysis, goniometry, optimization

## Abstract

The present study brings to attention a method to develop salicylic acid-based oil in water (O/W) microemulsions using a tensioactive system based on Tween 80, lecithin, and propylene glycol (PG), enriched with a vegetable oat oil phase and hyaluronic acid. The systems were physically characterized and the Quality by design approach was applied to optimize the attributes of microemulsions using Box–Behnken modeling, combined with response surface methodology. For this purpose, a 3^3^ fractional factorial design was selected. The effect of independent variables namely X1: Tween 80/PG (%), X2: Lecithin (%), X3: Oil phase (%) was analyzed considering their impact upon the internal structure and evaluated parameters chosen as dependent factors: viscosity, mean droplet size, and work of adhesion. A high viscosity, a low droplet size, an adequate wettability—with a reduced mechanical work—and clarity were considered as desirable for the optimal systems. It was found that the optimal microemulsion which complied with the established conditions was based on: Tween 80/PG 40%, lecithin 0.3%, oat oil 2%, salicylic acid 0.5%, hyaluronic acid 1%, and water 56.2%. The response surface methodology was considered an appropriate tool to explain the impact of formulation factors on the physical properties of microemulsions, offering a complex pattern in the assessment of stability and quality attributes for the optimized formulation.

## 1. Introduction

Salicylic acid (2-hydroxybenzoic acid) [1], known as a β-hydroxy acid compound, with low solubility in water [2], represents an active pharmaceutical ingredient with important roles in cosmetics and dermatologic treatment of acne [3], psoriasis [4], actinic keratosis [5] or warts [6], as a function of concentration. Thus, in concentrations between 0.5–2%, it is preferred in anti-acne formulations exerting keratoplastic and keratolytic effects [7]. Pronounced keratolytic effects are wielded in a concentration domain of 2–10% [8], while concentrations up to 30% are used in peeling procedures [9]. Acne represents a dermatologic condition with an inflammatory component, targeting sebaceous glands [10,11]. Its multifactorial character and high recurrence among adolescents, and also adult patients, suggest acne is a complex pathology [11,12]. Tissue disorder, which collapses with the development of scarring, is the result of the simultaneous action of hormonal, immunological, microbiological, and genetic factors [13]. Salicylic acid has been approved since 1985 by the FDA and is noted in the Federal Register as an active compound which can be included in over-the-counter topical formulations for acne treatment purposes, similar to resorcinol, resorcinol monoacetate, and sulfur [7]. Even if its application as an adjuvant in anti-acne formulations recommend salicylic acid as a traditional molecule [14], new insights and positive attributes tend to elicit interest concerning its involvement in curing acne, cellular mechanisms, or new formulations with targeted action. Salicylic acid is known to possess anti-inflammatory effects which are explained by mechanisms such as inhibition of prostaglandin synthesis, decreasing cyclooxygenase, and lipoxygenase release in tissues [15]. Due to its lipophilicity, salicylic acid can diffuse in deeper layers of skin in the pilosebaceous follicle, promotes lysis of desmosomes, and contributes to regeneration processes, especially when penetration enhancers are selected, as reported by Zhao Q. et al. [16]. Formulated in classical skin care products such as lotions [17], or ointments [18], it tends to localize in the upper layers of the skin, offering in this case a superficial exfoliating effect. Current interests highlight the importance of targeted therapy as a way to overcome the diffusional barrier of stratum corneum to promote a deeper localization of lipophilic compounds and access the affected sites [19]. Recent advances have revealed strategies to improve salicylic acid action at skin level and alleviate its irritative potential, and here can be classified a number of modern colloidal systems, namely: nanoparticles [20,21], nanoemulsions [19], and microemulsions [22]. Older studies reported also the possibility to encapsulate salicylic acid in liposomal formulations [23], hydrogels, or lipogels [18].

The study of microemulsions remains of outstanding interest in the pharmaceutical domain, with the aim of discovering new formulations that can increase the bioavailability of drugs in both topical and systemic routes [24]. Microemulsions are nanostructured carriers which can be designed to optimize the release of active substances on the skin site [25]. In the last decade, the versatility of microemulsions was highly appreciated due to their capacity of drug entrapment in nanosized form and implication as promotors for nanoparticle synthesis [26]. A large series of studies was proposed in order to target the skin route, highlighting major advantages [24], such as ease of preparation, the high power of drug solubilization, targeted therapeutic action, lack of drug side effects, and stability over time. Here it can be noted microemulsions with anti-inflammatory activity [24] antifungal [27], antipsoriatic [28], immunomodulatory [29], anticancer [30], antiacne [11], or regenerative therapeutic activity. The water titration method constitutes a simplified approach applied to develop in a feasible manner isotropic mixtures of oil, surfactant, cosurfactant and water in different proportions [31]. These systems are characterized by high thermodynamic stability [32] and a good penetration ability for skin delivery purposes [22,25]. Fluid O/W systems with low oil content are considered suitable for application on acne-prone skin which is subjected to an increase in sebum secretion. To exemplify this, Hung W.H. et al. established the use of a maximum oil concentration of 10% for anti-acne microemulsions [33]. Inclusion of calculated amounts of surfactant and cosurfactant is related to two main attributes—promotion of high solubilization of the oil phase and active substances, and a decrease in the interfacial tension at the O/W interface up to ultralow values. Thus, microemulsions are considered nanocolloids with particle dimensions of between 5–100 nm [34,35]. In the same manner, the stabilizers function as skin penetration enhancers, implied in lipid fluidization, and temporarily disrupt effects on lipidic membrane domains, assuring accumulation of a higher concentration of the active pharmaceutical ingredient (API) in dermis structures [36,37]. Those qualities were appreciated in several new studies designed to assure superior therapeutic effects for azelaic acid [33], adapalene [38], nicotinamide [39], or fusidic acid [40].

It is noteworthy that an increased interest in vegetable oil selection was observed in the field of microemulsion preparation [22,28,41]. Oat oil represents a lipophilic phase extracted from *Avena sativa* sp., which is appreciated for its antioxidant activity [42] and recognized for its value in the process of ceramide synthesis at the skin level [43]. Limited studies were found in the favor of oat oil inclusion as a part of colloidal dispersions. In the US 5620692 patent, the use of oat oil was emphasized in cosmetic and dermatologic formulations on a concentration domain of 5–10%. The study recommended it as a biocompatible and potent antioxidant at skin level [44].

Tween 80 is one of the most widely selected non-ionic surfactants in microemulsion design, being generally recognized as safe for topical delivery and suitable for O/W formulations. Associated with propylene glycol as a cosurfactant, the solubilization effect, and the penetration activity through internal skin layers was enhanced as reported in previous studies [45,46]. It has been stated that the inclusion of propylene glycol as cosurfactant will assure an increase in the systems` stability, expanding the area of microemulsion on the pseudoternary diagrams [45]. In a recent study of de Sá A.Á.M. et al., the selection of Tween 80/PG in a ratio of 2:1 from 10% up to 30% was highlighted. This mixture was suitable for stabilizing O/W microemulsions with essential oils of 2–5% and an oil phase of 20–30% as potential skin lightening agents [47].

The addition of lecithin as a second surfactant in small concentrations may open valuable perspectives in the design of O/W microemulsions [48]. Lecithin represents a complex product (based on acetone insoluble phosphatides, triglycerides, and fatty acids), with a large utility in the food industry and in pharmaceutical and related biomedical fields due to its biocompatibility and similarity with the cellular phospholipids of living organisms [49]. Several sorts of lecithin were reported in the literature as valuable for micro-/nanoemulsion design, either alone or in combination with other stabilizers: granular de-oiled soybean lecithin (Epikuron 200) [48], Lipoid S75 (soybean phospholipids with 75% phosphatidylcholine) [50], soybean lecithin with 99% phospholipids [51], granular soybean based L-α-lecithin [52,53]. Selected in small concentrations up to 1%, lecithin represents a natural and biocompatible component with a double function: capacity for oil solubilization and the promotion of hydrating effects. A recent study regarding the development of another group of nanocolloids, called nanoemulsions, proposed the use of lecithin 1% in association with small concentrations of Tween 80 to promote O/W systems [50]. Nanoemulsions have a similar composition to microemulsions, but reduced levels of surfactant and cosurfactant are required. In this case, thermodynamically unstable systems are generated. Paradoxically, the nomenclature of micro-/nanoemulsions generates soft polemics at first sight, so it does not indicate the real dimension of particles dispersed in these systems. If microemulsion particles are considered to reach 5–100 nm after spontaneous emulsification, in the case of nanoemulsions, distinctive aspects may be exposed. The particles’ dimensions tend to reach from 200 nm up to 500 nm, depending on the external forces implied in the emulsification process and the type of the stabilizers [54].

For topical vehicles with acne alleviation purposes, the use of functional polymers, such as hyaluronic acid, will assure optimal hydration during the period of treatment, protecting the skin against potential irritative effects caused by anti-acne molecules [55]. Alkrad J.A. et al. proposed W/O microemulsions with isopropyl palmitate as a continuous phase to sustain hyaluronic acid delivery on the skin site. A low-molecular-weight hyaluronic acid (22 kDa) of 2% was chosen as a hydrophilic molecule dispersed in an oil-based vehicle that can surpass stratum corneum and promote protective action [56]. Thereby, in an O/W system, low concentrations of hyaluronic acid will be solubilized in water phase and will only assure hydration in superficial layers of the skin, without passing the skin barrier.

In the light of these findings, the aim of the study was to develop microemulsions with salicylic acid as a therapeutic solution in acne alleviation. We proposed O/W fluid systems, stabilized with a biocompatible mixture of Tween 80/lecithin/propylene glycol, previously reported in our preliminary work [57]. Granular l-α-lecithin with more than 97% phospholipids was selected as a second surfactant as previously reported [53]. The stabilizer mixture, selected at a concentration domain of 20.1–40.5%, can successfully solubilize an oil phase of 1–2% and incorporate salicylic acid 0.5%. Hyaluronic acid 1% was added to enrich the quality profile of the microemulsions. The experimental background proposed an inspection of the physical properties of the microemulsions, concerning pH analysis, conductivity analysis, refractive index tests, rheological evaluation, the study of particle behavior, including here dynamic light scattering evaluation and zeta potential analysis. A goniometric study completed the discovery of the internal properties of the systems, emphasizing superficial and adhesion phenomena.

Thereby, the desired micro emulsified system was considered to comply with several critical quality attributes of high significance for topical application. In this way, the study will be further focused on discovery of one or more systems characterized by high viscosity, low droplet size, and an adequate wettability. Viscosity represents the main physical parameter studied to assess the flow of behavior of microemulsions and obtain preliminary information related to the spreading effect on tissue. According to the work of Benigni M. et al., a system with high viscosity is preferred instead of a fast-flowing product [58]. Viscosity is one of the parameters that influence not only the quality of the final system but also the bioavailability of API in the tissues [59]. On the other hand, the obtaining of nanosized droplets dispersed in the aqueous vehicle will contribute to the adhesion process due to an increase in the surface area determined by the surfactant/cosurfactant mixture (S/CoS) implied in the stabilization [60]. Adhesion work was studied as a parameter that can connect viscosity and droplet size together. These parameters were further analyzed by establishing associations with the influence of the main formulation factors. The research of superficial properties of microemulsions will define dynamic processes related to the systems’ behavior as they come into contact with a surface [61].

From a qualitative point of view, we suggested clarity as a reference parameter in the depiction of the optimal microemulsion. The application of a transparent formulation on the skin site can offer a pleasant experience, without leaving colored traces. Its elegant appearance, completed by appropriate pharmaceutical attributes increases confidence and adherence in affected patients [33].

Pharmaceutical Quality by design (QbD) defines a rigorous concept that sustains the integration of quality into a product from the early stages of development, and is found throughout the entire cycle of development, fabrication, and regulation of a medicine. The study of critical quality attributes is rallied to a desired therapeutic effect centered on patient, and it imposes a connection with critical material attributes and critical parameters of processes that need to be optimized [62,63]. A complex interpretation of experimental results and an accurate discovery of optimal formulations is based on mathematical modeling which combines mathematical principles, statistics, and graphical analysis. As part of the Quality by design concept, the design of the experiment (DOE) represents a far-reaching method which deals with a variate panel of techniques that can be applied in order to optimize new pharmaceutical formulations [64,65] or processes [66]. Thus, several techniques can be mentioned, particularly response surface methodology (RSM) which encompasses statistical analysis of response, factorial plotting, contour and surface plotting, with the possibility of assessing predictive responses based on the response optimizer tool [67,68]; subsequently, alternative practical analyses such as Mixture design [69] and the Taguchi approach [70,71] can also be implemented. Mathematical modeling was considered a useful tool to explain the impact of formulation factors on the physical properties of microemulsions, as previously reported [19,21,67].

In our case, the Quality by design approach was applied to optimize the physical quality attributes of microemulsions. A three-factor, three-level fractional factorial design was chosen by creating a Box–Behnken model to study the response of three formulation factors and applying RSM. Thus, the effect of the independent variables, namely X1—Tween 80/propylene glycol (%); X2—lecithin (%); and X3—oil phase (%) was explored considering their impact upon internal structures and the following dependent variables—Y_1_: viscosity (cP), Y_2_: mean droplet size (nm), and Y_3_: work of adhesion (mN/m).

## 2. Materials and Methods

### 2.1. Materials

All the actives and reagents used in the study were of analytical grade. Salicylic acid was purchased from Chemical Company (Iaşi, Romania). Tween 80 was purchased from Carl Roth GmbH + CoKG (Karlsruhe, Germany), while granular lecithin (l-α-lecithin) from soybean oil (with ≥97% phospholipids) was supplied from Acrōs Organics (Thermo Fischer Scientific, Waltham, MA, USA). Propylene glycol was acquired from Sigma Aldrich (Taufkirchen, Germany). Hyaluronic acid low molecular weight (LMW, 10–200 kDa) was purchased from Elemental (Oradea, Romania), and oat oil from Aroma Zone (Cabrieres-d’Avignon, France). Ultrapure distilled water with a specific resistance of 18.2 MΩ/cm, and total organic carbon (TOC) of less than 5 μg/L was generated from a Milly-Q^®^ Direct 8 Water Purification System (Merck Millipore, Bedford, MA, USA), and used as aqueous phase.

### 2.2. Preparation of Salicylic Acid-Based Microemulsions

Microemulsions with salicylic acid were developed using a mixture of Tween 80/PG with good solubilization properties. Thirteen microemulsions, denoted as MELSA 1–MELSA 13, were generated by Minitab software and represented as a 3^3^ fractional factorial design. The systems were formulated by applying the water titration method, under continuous stirring at 70 °C. The use of heat at the moment of preparation was considered favorable to assure the homogenization of microemulsions. Initially, calculated amounts of lecithin were weighed and placed in a melting pot, on a hotplate stirrer (DLAB MS-H380-Pro, DLAB Scientific, Beijing, China). Based on the affinity of lecithin for the oil phase, at the melting point of lecithin, the proper amount of oat oil was added, resulting in an orange-like lipophilic mixture. Tween 80 and PG were separately mixed to solubilize salicylic acid, and finally added drop by drop into the lipophilic phase by stirring. In the last step, hyaluronic acid was solubilized in 8 mL of distilled water and then embedded in the above-mentioned mixture of lipophilic phase and surfactants. After a proper homogenization, the water titration method was initiated, keeping operational conditions constant, for up to 2 h. Heating was gradually reduced to avoid evaporation. Clear or almost translucent microemulsions were obtained and placed into equilibration in a calm environment.

The composition of microemulsions with salicylic acid MELSA 1–MELSA 13, designed according to Box–Behnken factorial matrix, is further presented in Table 1, and corresponds to the final formulations.

**Figure 1:**
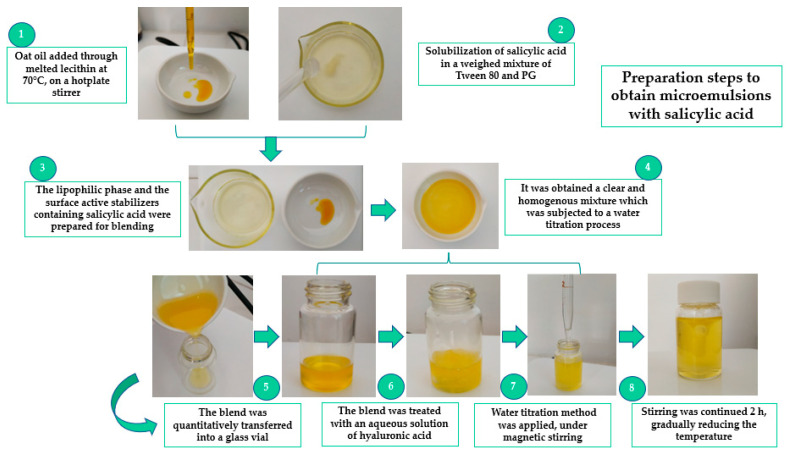
Critical steps performed in the preparation process of microemulsions with salicylic acid.

Figure 1 shows a rigorous representation of the critical steps adopted in the process of microemulsion preparation.

The method of preparation was based on the aqueous titration method, under magnetic stirring at 900 rpm. It was adapted to the experimental conditions, including in this case, a short period of heating, under a controlled temperature, at 70 °C. Chen L. et al., reported the use of heating in their preparation step to support a better homogenization of samples [72]. In such a way, in our study, heating was helpful to assure the melting of lecithin and its homogenization with the oil phase and further components. Heating was gradually reduced to avoid evaporation or unexpected degradative reactions of the components which might have been detrimental for the evaluation process [73].

Tween 80 (Polysorbate 80) represents a non-ionic emulsifier commonly selected in the formulation process of nanodispersions due to its safety profile. Characterized by a Hydrophilic Lipophilic Balance value (HLB) of 15, Tween 80 was selected in the formulation process of O/W microemulsions. Its emulsifying properties are mainly attributed to a balanced chemical structure based on a polyethoxylated sorbitan ring (with hydrophilic effects), grafted with a long C_18_-monooleate chain responsible for interaction with lipophilic entities [61]. In association with a medium-chain alcohol, in our case, propylene glycol is formed as a binary mixture [74] with a powerful solubilization effect for the oil phase and lipophilic API. It is well known that Tween 80 and propylene glycol are implied in penetration activity at the level of stratum corneum, ensuring the diffusion of the API through deeper layers of the skin [36,75,76]. Several studies have concentrated on this phenomenon, and proposed Tween 80/PG tensioactive blend to sustain O/W microemulsion generation [46,77,78].

Taking into account the solubility parameter, salicylic acid has a low solubility in water at room temperature (1 g/460 mL) [79]. In our study, the API was solubilized in a mixture of Tween 80/PG (2:1), in a total weight of 20–40% from the final 20 mL of the sample. The final concentration of salicylic acid in microemulsion was 5 mg/mL. Thereby, the quantity of salicylic acid from our microemulsions cannot be solubilized with 56.2–77.2% of the included water.

Several studies reported the use of alcohol-based solvents, polysorbates and their binary mixtures to solubilize salicylic acid [80,81,82]. Solubility studies were conducted by applying Van’t Hoff equation [80,81]. Ethanol presented a good solubilization power for salicylic acid, expressed as 325.38 mg/mL at room temperature (298 K) [80]. However, the use of ethanol in topical products is usually substituted by other biocompatible solubilizers as a result of its irritative potential. As an example, for propylene glycol, the solubility of salicylic acid was determined to be 221 mg/mL (1.59 M). A similar solubilization profile was obtained for the binary mixture of propylene glycol/ethanol (1.59 M) [81]. Hence, for 100 mg of salicylic acid required in our formulation, it can be stated that 0.452 mL of PG is necessary to promote solubilization at 25 ± 0.5 °C. In our formulations, PG was selected between 6.65–13.35% (1.33–2.67 mL) and contributed to the solubilization process together with Tween 80.

From another perspective, the aqueous phase can diminish the solubilization power of a binary mixture of Tween 80/PG, as a function of its concentration. An older study was based on a solubility screening of salicylic acid, using Tween 80 as a solvent, in combination with other co-solvents. Water titration was applied to obtain the critical miscibility ratio of salicylic acid/Tween 80 in water. During the analysis, it was observed that PG did not affect the solubility property of Tween 80. In addition, it was graphically noted that a concentration of Tween 80 up to 60% in an aqueous solution was able to solubilize a high concentration of API up to 20% [82]. Paying attention to these findings, it can be appreciated that 0.1 g of API can be successfully solubilized by a Tween 80/PG mixture of 20–40%.

To ensure a high safety profile, soybean lecithin was considered for integration as a natural second surfactant. It has an HLB of 5, being selected in large concentrations to form W/O dispersions. However, in small concentrations of 0.1–0.5%, lecithin can enhance the quality profile of microemulsions without hampering HLB value of the systems. In the study of Špaglová M. et al., topical microemulsions were formulated using a reduced level of Tween 80 of 26.5% and isopropyl alcohol of 26.5%. Lecithin was selected at 0.75% and was considered one of the formulation factors that influenced the behavior of microemulsions in the process of diffusion [83].

According to a previous study regarding microemulsion preparation and characterization, hydrophilic lipophilic balance values required to attain stable systems (HLB_ME_) were calculated. The HLB_ME_ required was considered, taking into consideration the HLB_Tween 80_ = 15 and HLB_Lecithin_ = 5. The following formula (Equation (1)), proposed by de Melo Cotrim A.C. et al. was applied [84]:

(1) $${\mathrm{HLB}}_{\mathrm{ME}}{=[(\mathrm{HLB}}_{\mathrm{Tween}\;80}\;\cdot {\;F}_{\mathrm{Tween}\;80}{)+(\mathrm{HLB}}_{\mathrm{Lecithin}}\;\cdot {\;F}_{\mathrm{Lecithin}}{)]/(10\;-\;F}_{\mathrm{PG}}),$$

where $F_{\mathrm{Tween}\;80}$ represents Tween 80 fraction, ${\;F}_{\mathrm{Lecithin}}$—lecithin fraction, $F_{\mathrm{PG}}$—propylene glycol fraction, and $F_{\mathrm{Tween}\;80}$ + ${\;F}_{\mathrm{Lecithin}}$ + $F_{\mathrm{PG}}$ = 10, by reporting to the total mass of the three solubilizers selected in each formulation.

### 2.3. Organoleptic Analysis

Based on visual inspection of the obtained microemulsions, important clues were observed, including aspect, color, odor, involvement of phase separation, and other instability phenomena, which were correlated with their composition. The organoleptic analysis was considered over a period of one month.

### 2.4. pH Determination

The pH of each formulation was measured using a Mettler-Toledo seven compact pH-meter (Mettler-Toledo GmbH, Greifensee, Switzerland), equipped with a silver-based pH glass electrode proper for small samples. Before each measurement, calibration was performed using buffer solutions of pH 7 and pH 4. Measurements were performed in triplicate, at 25 ± 0.5 °C.

### 2.5. Conductivity Analysis

The phase behavior was inspected along with the influence of formulation factors for each sample at 25 ± 0.5 °C, using a Corning 441 bench conductivity-meter (Cole Parmer Instrument Company, LLC, Vernon Hills, IL, USA). The measurements were recorded in triplicate.

### 2.6. Refractive Index Determination

The refractive index was measured at 25 ± 0.5 °C for each microemulsion, proving their clarity and isotropic nature. The determinations were performed using a Krüss DR 201-95 digital refractometer (Krüss Optronic GmbH, Hamburg, Germany). Distilled water with a refractive index of 1.3330 was used as a reference standard for calibration before each test. The measurements were recorded in triplicate.

### 2.7. Rheological Evaluation

A rheological evaluation was performed for each microemulsion, with respect to phase behavior and flow characteristics as a function of internal composition based on the formulation factors involved. MELSA 1–MELSA 13 microemulsions were analyzed at 25 ± 0.5 °C, using Multi Visc Rheometer (Fungilab, Barcelona, Spain), equipped with an LCP spindle, as previously reported [57,85]. Viscosity measurements were performed by applying a rotation speed between 0.3–60 rpm, corresponding to a shear rate between 0.36 and 73.38 s^−1^.

### 2.8. Droplet Size and Zeta Potential Analysis

Droplet size distribution and polydispersity index (PDI) were studied at 25 ± 0.5 °C, applying a dynamic light scattering (DLS) technique on undiluted samples. Accurate measurements were performed using a VascoKin particle analyzer (Cordouan Technologies, Pessac, France), equipped with a 638-nanometer laser. In addition, the study of particle behavior was undertaken using an in-situ head which assured a back-scattering mode, by applying a scattering angle of 170°. Size distribution (nm) as a function of intensity (a.u.) was obtained for each microemulsion sample, according to the Cumulant algorithm, being associated with the autocorrelation function expressed as time (μs) as a function of intensity (a.u.). The results were fitted using the Rayleigh model [86,87].

Estimation of droplet size was performed with respect to the presence of spherical particles dispersed in a Newtonian fluid, by applying the Stokes–Einstein equation, as it can be seen in Equation (2) [73,86]:

(2) $$d_{H\;\mathrm{app}\;}{=k}_{B}{T/3\pi \eta D}_{\mathrm{app}}{,}_{\;}$$

where $d_{H\;\mathrm{app}\;}$ represents the hydrodynamic diameter, $k_{B}$—Boltzmann constant, T—the absolute temperature, η—the viscosity of the medium, and $D_{\mathrm{app}}$—the apparent diffusion coefficient estimated from the autocorrelation function.

The Zeta potential was studied on the basis of laser doppler electrophoresis technology [88]. Determinations were performed using the Wallis Zeta potential analyzer (Cordouan Technologies, Pessac, France), equipped with a 20-mW diode laser source with a wavelength of 635 nm. For both analyses, calibration was made using a standardized colloidal dispersion of latex of 1% with particles of 100 nm. The results were presented as the mean values of three determinations for each tested sample.

### 2.9. Superficial Analysis

The depiction of superficial properties of microemulsions was based on the measurement of free superficial energy and contact angle, using CAM-101 Goniometer, equipped with a Hamilton syringe, a C209-30 needle, and a digital camera (KSV Instruments Ltd., Espoo, Finland), as reported in [89,90]. Drops of each sample (μL) were applied on microscope slides, captured with a digital camera, and measured over a period of 64 ms by an automated curve-fitting program. Young equation (Equation (3)) was fitted with two models of analysis: the pendant drop and contact angle models [85]:

(3) $$\gamma_{\mathrm{SG}}{=\gamma }_{\mathrm{SL}}{+\gamma }_{\mathrm{LG}}\cos\theta ,$$

where $\gamma_{\mathrm{SG}}$ represents the interfacial tension to solid/gas (S/G) interface, $\gamma_{\mathrm{SL}}$— interfacial tension to solid/liquid (S/L) interface, $\gamma_{\mathrm{LG}}$—superficial tension to liquid/gas (L/G) interface, and θ—the contact angle made by the liquid drop with the solid surface, which gave information concerning the wettability of microemulsions.

In addition, work of adhesion (W) was determined for each system, considering the Dupré equation (Equation (4)), which can be applied in accordance with the results obtained after contact angle analysis [91]:

(4) $${W=\gamma }_{\mathrm{LG}}(1+\cos\theta ),$$

where, W represents the work of adhesion, $\gamma_{\mathrm{LG}}$—the superficial tension to L/G interface, and θ—the contact angle determined previously, by applying the contact angle model. All determinations were performed in triplicate at 25 ± 0.5 °C.

### 2.10. Data Analysis and Optimization of Microemulsions Using Box–Behnken Design

All the experiments were performed after one month of preparation. The data obtained for the experiments were expressed as mean values ± standard deviation (SD), using Microsoft Excel (Microsoft, Redmond, WA, USA). A single factor ANOVA test was applied to assess statistically significant differences between formulations in the case of a superficial analysis. An ANOVA test for the optimization process was performed using Minitab software. A probability of 0.05 with *p* < 0.05 was considered as the level of significance.

To optimize the formulation parameters and find an optimal microemulsion with proper physical and quality characteristics, three independent variables were selected, namely: Tween 80/PG concentration (X_1_), lecithin concentration (X_2_), and oat oil concentration (X_3_), at three levels of variation, coded as follows: low (−1), medium (0) and high (+1). A 3^3^ fractional factorial design was generated using Minitab software (Minitab-Trial version, LLC, State College, PA, USA). Overall, 13 formulations equivalent to 13 runs with one center point without replication were generated, developed, and evaluated, choosing several responses as dependent variables, coded as Y: viscosity (Y_1_), mean droplet size (Y_2_), and work of adhesion (Y_3_).

For each dependent variable, the influence of formulation factors (X_1_, X_2_, and X_3_) was represented using a quadratic model quantified by the following fit of second order polynomial equation (Equation (5)), generated by software:

(5) $$Y=\beta +\;\beta_{1}X_{1}+\beta_{2}X_{2\;}+\beta_{3}X_{3}{+\beta }_{11}X_{1}^{2}+\;\beta_{22}X_{2\;}^{2}+\beta_{33}X_{3\;}^{2}+\beta_{12}X_{1}X_{2}+\;\beta_{13}X_{1}X_{3}+\;\beta_{23}X_{2}X_{3},$$

where Y represents the response, $X_{1}$–$X_{3}$—the linear terms, $X_{1}^{2}$–$X_{2\;}^{2}$– the quadratic terms, $X_{1}X_{2}$, $X_{1}X_{3}$, $X_{2}X_{3}$—the interaction terms, β—the free coefficient or intercept, $\beta_{1}$–$\beta_{3}$—the coefficients of linear terms, $\beta_{11}$–$\beta_{33}$—the coefficients of quadratic terms, and $\beta_{12}$, $\beta_{13}$, $\beta_{23}$—the coefficients of interaction terms of the model.

The equations were interpreted using ANOVA analysis. As a part of the surface response methodology, Pareto charts were suggested as valuable representations to determine which of the independent factors were significantly determinative of the evaluated parameters. The predicted response values for optimal formulations were assessed and compared with the experimental results based on factorial plotting of the main effects of variables and the response optimization approach [92].

## 3. Results

### 3.1. Formulation Design

The microemulsion design was based on the development of a 3^3^ fractional factorial plan, using three formulation factors as independent variables, and implementing a proper preparation protocol based on water titration. In this way, three independent variables, denoted as X_1_, X_2_, and X_3,_ are presented in Table 2, considering three levels of variation: Low (−1), Medium (0), and High (+1). Table 3 provides the experimental matrix generated from the software.

The organoleptic characteristics of microemulsions were assessed including their aspect and color, odor, and the presence of instability phenomena over time. The formulations were inspected after a period of one month and then characterized. Their clarity after one month, specified in Table 3, was graded with three symbols as a function of the following three clarity parameters: “+” was checked for opalescent systems, “++” was checked for systems with low clarity, and “+++” was attributed to systems with high clarity. Subsequently, HLB required values were calculated for each microemulsion. It can be seen that lecithin and PG inclusion, in limited concentration, did not affect HLB_ME_. The values were close to the HLB_Tween 80_ value, which signifies that the designed mixture promoted stability for the microemulsion-based samples.

**Table 3:** Experimental matrix of microemulsions MELSA 1–MELSA 13.

Formulation	Run Order	^1^ Clarity	Tween 80/PG	Lecithin	Oil	HLB_ME_
MELSA 1	1	++	−1	−1	0	14.90
MELSA 2	2	+++	+1	−1	0	14.46
MELSA 3	3	++	−1	+1	0	14.62
MELSA 4	4	+++	+1	+1	0	14.80
MELSA 5	5	+++	−1	0	−1	14.75
MELSA 6	6	+++	+1	0	−1	14.87
MELSA 7	7	+	−1	0	+1	14.75
MELSA 8	8	+++	+1	0	+1	14.87
MELSA 9	9	+++	0	−1	−1	14.93
MELSA 10	10	+++	0	+1	−1	14.72
MELSA 11	11	+	0	−1	+1	14.93
MELSA 12	12	+	0	+1	+1	14.72
MELSA 13	13	+++	0	0	0	14.85

^1^ Notation of clarity parameters: “+” was attributed for an opalescent system, “++”—for a low clarity system, and “+++”—for a high clarity system.

**Figure 2:**
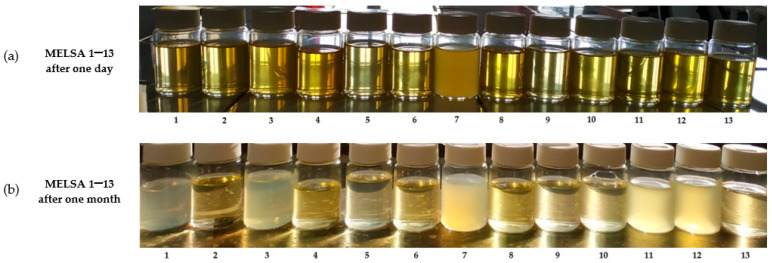
Photo capture of MELSA 1–MELSA 13 microemulsions at one day after preparation—case (**a**), and after one month—case (**b**), visualized at room temperature, 25 ± 0.5 °C.

On the first day after preparation, all microemulsions with the exception of MELSA 7 had a clear appearance. Their color and odor were associated with the presence of Tween 80, lecithin, and the oil phase, resulting in systems with a yellowish aspect, with a variation in intensity depending on the concentration of the components. In the case of MELSA 7, the minimum concentration of 20.3% Tween 80/PG/lecithin and 2% oat oil determined the formation of an opalescent system. In Figure 2—case (a), a photo capture of microemulsions after one day from preparation at 25 ± 0.5 °C is presented. In the first month, four more microemulsions tended to vary in opalescence, namely: MELSA 1, MELSA 3, MELSA 11, and MELSA 12, which can be observed in a photo capture of microemulsions after 1 month, presented in Figure 2—case (b) at 25 ± 0.5 °C. The opalescence was persistent due to a medium concentration of oil at 1.5%, dispersed with minimum concentrations of Tween 80/PG/lecithin at 20.1% (in the case of MELSA 1), or 20.5% (in the case of MELSA 3). On the other hand, the use of a maximum oil level of 2% affected the clarity of MELSA 11 and MELSA 12 even if Tween 80/PG/lecithin were set at medium levels of 30.1% or 30.5%.

The selection of the maximum level of Tween 80/PG of 40%, associated with lecithin 0.1%, 0.3%, or 0.5% was favorable to assure a complete dispersion of the oil phase at 1%, 1.5%, or 2%, resulting in clear systems.

### 3.2. pH Determination

For the tested microemulsions, the pH was placed in the near physiological range, which is 4–6 for topical formulations. The measured values corresponded to an acidic pattern, being placed in the following domain: 3.48 ± 0.01–3.71 ± 0.01, and presented in Table 4. The values were appropriate for a topical product recommended in the treatment of acne-affected skin. It can be stated that the pH was influenced by the microemulsions’ composition, being divided into three groups of values as a function of water content and the inclusion of Tween 80/PG. At concentrations of Tween 80/PG of 20%, and a large amount of water (77.20–76.20%), the pH was 3.48 ± 0.01–3.50 ± 0.01. The increase of the Tween 80/PG amount at the intermediate level of 30%, with a smaller water content of 66.00–67.4%, influenced the pH to reach values of 3.53 ± 0.01–3.59 ± 0.01. Finally, the maximum amount of Tween 80/PG of 40%, and a reduced water content of 56.20–57.20%, was proper for pH values between 3.63 ± 0.02–3.71 ± 0.01. The presence of lecithin and oat oil was not significant for the elevation of pH (*p* > 0.05).

### 3.3. Conductivity Analysis

**Figure 3:**
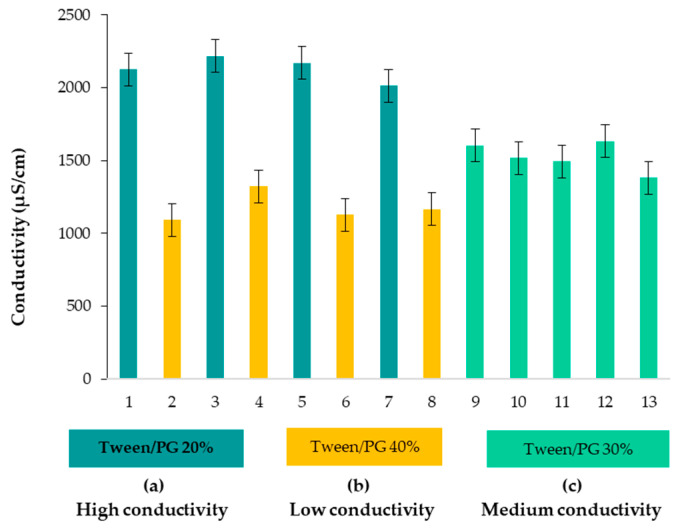
Graphical representation of conductivity values for microemulsions, dividing them as systems with (**a**) High conductivity, (**b**) Low conductivity, and (**c**) Medium conductivity, depending on Tween 80/PG concentration.

Considering the conductivity parameter, microemulsions were placed in the group of oil in water (O/W) formulations. The values are shown in Table 4. High values of conductivity, placed in the following domain of 506.0 ± 3.0–1088 ± 1.7 μS/cm, were correlated with the main factors of Tween 80/PG concentration and water concentration. Lecithin and oil implications in conductivity variation were insignificant (*p* > 0.05). A suggestive representation of conductivity values for each microemulsion (1–13) was proposed in Figure 3, placing them in three categories, denoted as (a), (b), and (c). Thus, dividing the results into three groups, as a function of Tween 80/PG concentration, it can be stated that high values of conductivity (986.3 ± 1.5–1088.0 ± 1.7 μS/cm) were attributed to systems with a high content of water of 76.20–77.20%, and Tween 80/PG of 20%. At intermediate levels of Tween, 80/PG of 30% and a decrease in water content of 66.00–67.40% was appropriate for conductivity values between 661.3 ± 1.5–786.0 ± 0.0 μS/cm. Following the third group of microemulsions, the values of conductivity were placed between 506.0 ± 3.0–621.3 ± 0.6 μS/cm. These values were attributed to the maximum level of Tween 80/PG of 40%, associated with a minimum content of water of 56.20–57.20%.

### 3.4. Refractive Index Determination

**Figure 4:**
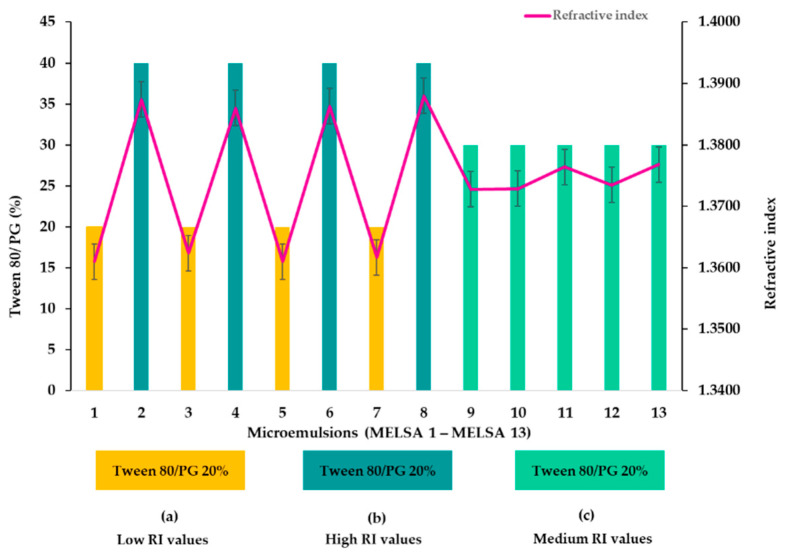
Graphical representation of refractive index variation for microemulsions, considering three cases: (**a**) systems with low RI values, (**b**) systems with high RI values, and (**c**) systems with medium RI values, depending on Tween 80/PG concentration.

The isotropic nature of the tested microemulsions was confirmed after refractive index (RI) determination. All the results are presented in Table 4. The refractive index variation is presented in Figure 4, where a refinement of the results is suggested, considering the impact of Tween 80/PG concentration. Herein, it can be observed that at a minimum concentration of Tween 80/PG of 20%, RI varied between 1.3610 and 1.3624—group (a). In group (c), specific for microemulsions with Tween 80/PG of 30%, RI varied between 1.3728 and 1.3768. In the last case, for microemulsions with Tween 80/PG of 40%, specific for group (b), the refractive index varied between 1.3860 and 1.3880. The lecithin and oil phase variation did not affect the refractive index parameter and their influence was considered insignificant (*p* > 0.05).

### 3.5. Rheological Evaluation

**Figure 5:**
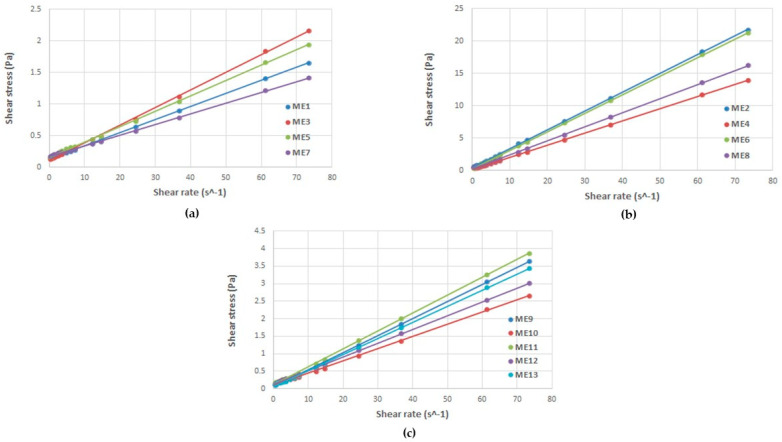
Rheological profiles expressed as shear stress as a function of shear rate for MELSA 1–MELSA 13 microemulsions, tested at 25 ± 0.5 °C, proving their Newtonian behavior, divided in three groups: (**a**) microemulsions with low viscosity, (**b**) microemulsions with high viscosity, and (**c**) microemulsions with intermediate viscosity.

During viscosity measurements for MELSA 1–MELSA 13 microemulsions, it was demonstrated that Newtonian behavior was particular for this kind of fluid colloidal systems. In accordance with Newton’s Law, the viscosity parameter was maintained constant at shear stress and shear rate variation. Table 4 presents viscosity results (cP), obtained from the slope of the straight line after generation of regression equations specific for linear profiles of shear stress as a function of shear rate for each tested microemulsion. Determination coefficients (R^2^ = 0.9992–0.9999) showed a good fit for the model of viscosity. Consequently, in Figure 5, cumulative rheological plots of shear stress (Pa) as a function of shear rate (s^−1^) were provided to sustain Newtonian behavior of microemulsions tested at 25 ± 0.5 °C. For a better visualization, rheological profiles were divided in three groups depending on Tween 80/PG concentration and denoted as (a) microemulsions with low viscosity (formulated with Tween 80/PG 20%), (b) microemulsions with high viscosity (formulated with Tween 80/PG 40%), and (c) microemulsions with intermediate viscosity (formulated with Tween 80/PG 30%). As suggested by the rheological profiles, viscosity was expressed in Pa∙s and then converted into cP. The final results reported in Table 4 were used for interpretation and optimization of viscosity as a critical quality attribute.

As a result, viscosity values were placed between 16.94 ± 2.16–292.69 ± 4.18 cP and associated with the composition of the systems. Three groups of microemulsions, which can be characterized by taking into consideration the variation of Tween 80/PG concentrations, were found to be the main critical parameter for the flow behavior of the designed systems. Lecithin and oil phase amounts cannot be regarded as significant in this case (*p* > 0.05).

Consequently, systems with low, medium, and high viscosity were distinguished. The first group containing a low level of Tween 80/PG of 20% was characterized by low viscosities (16.94–27.89 cP), while in the second group, a medium level of Tween 80/PG of 30% determined a slight increase in viscosity (34.82–51.23 cP). By contrast, high values (188.17–292.69 cP) were found in the case of microemulsions with a maximum level of Tween 80/PG of 40%. It was presumed that elevation of Tween 80/PG at 40% determined a sharp increase in viscosity with positive outcomes for the quality profile of the systems, which will be further pointed out in the optimization process.

### 3.6. Droplet Size and Zeta Potential Analysis

For the study of particles and the assessment of internal structure data of microemulsions, a dynamic light scattering technique was applied. Mean droplet size (Ds) and polydispersity index (PDI) were measured. Stabilization processes and particular types of forces implied in particle dispersion were proposed after zeta potential (ζ) analysis. The results obtained during measurements at 25 ± 0.5 °C are presented in Table 4.

The data were processed using two models of analysis, specifically the Cumulant method and a distribution method based on Sparse Bayesian Learning (SBL) algorithm related with the rendering of a multimodal spreading of particle domains quantified as peaks on the graphical representation of scattering intensity as a function of droplet size. The most reliable model for our determinations was the Cumulant model related to the estimation of the mean hydrodynamic diameter (Z average). Consequently, mean droplet size was expressed as the average of the three evaluations. The values were estimated by the software and interpreted.

Overall, mean droplet size varied between 1.58 ± 0.20 nm and 37.72 ± 4.55 nm, being correlated with the composition of microemulsions. Ds was significantly influenced by Tween 80/PG (%) and oat oil (%) (*p <* 0.05). We observed that the selection of a narrowed domain of oil phase, in our case 1–2%, determined a fine dispersion of oil droplets responsible for the encapsulation of the lipophilic active compound. The oil particles were stabilized with a mixture composed of Tween 80, propylene glycol, and lecithin. Thereby, at a minimum concentration of oil phase and Tween 80/PG and a medium concentration of lecithin, the mean diameter of MELSA 5 was found to be 3.43 nm. An increase in diameter was observed for MELSA 1 when the oil level settled at 1.5% resulting in particles with a diameter of 11.35 nm. By modifying the lecithin level at 0.5%, the diameter was approximately doubled at 21.88 nm in the case of MELSA 3. In the same manner, the maximum hydrodynamic diameter of 37.72 nm was obtained for MELSA 7, and was justified by the selection of the highest amount of oil phase at 2%.

In the middle domain of mean droplet size corresponding to a level of Tween 80/PG of 30%, a better dispersion effect was obtained. Thus, at minimum and medium concentrations of 1–1.5% of the oil phase, Ds varied in a narrowed domain of 4.11–5.30 nm for MELSA 9, MELSA 10, and MELSA 13. The lecithin level did not induce a significant influence in droplet size in this case, but a high relevance can be observed for MELSA 11 MELSA 12 systems, formulated with oil phase of 2%. At a minimum level of lecithin of 0.1%, the Ds was 6.45 nm, while the switch to the maximum level determined a four-fold magnification of the diameter, attaining 26.02 nm.

Small values of Ds were obtained for microemulsions prepared with the maximum level of Tween 80/PG of 40%, in a domain of 1.58–5.88 nm. In this case, two groups of microemulsions can be distinguished. Focusing on MELSA 2 and MELSA 4 systems with a constant level of oil phase of 1.5%, the increase of lecithin level from 0.1% to 0.5%, determined a slight modification of Ds from 3.22 nm to 5.88 nm. Furthermore, balancing the variation rule in the favor of oil phase, and keeping a constant level of lecithin of 0.3%, Ds increased from 1.58 nm to 2.17 nm for MELSA 6 and MELSA 8, respectively. As a final remark in order to support the significance of formulation parameters, with respect to Ds it can be emphasized the following note that was precise for the optimization step: the higher the Tween 80/PG concentration, the smaller became the Ds, with good results at the selection of oil phase at the maximum level.

**Figure 6:**
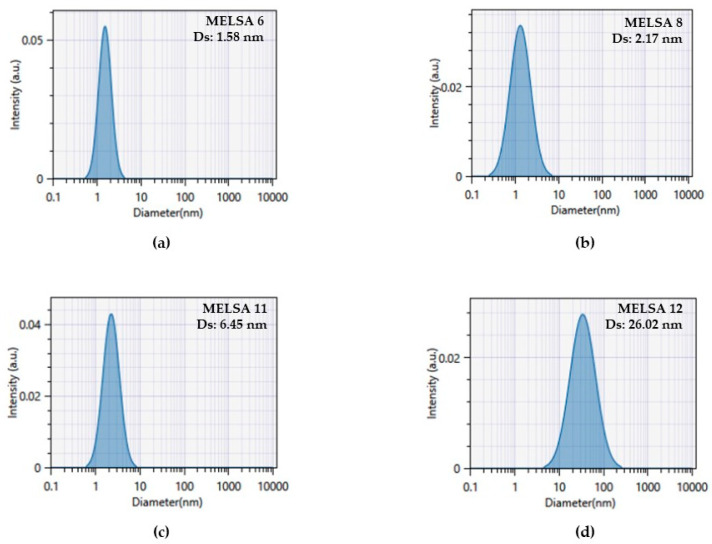
Cumulant profiles of scattering intensity as function of droplet size for the most representative systems emphasized in optimization process: (**a**) MELSA 6, (**b**) MELSA 8, (**c**) MELSA 11 and (**d**) MELSA 12.

In Figure 6, profiles of scattering intensity as a function of droplet size are presented, in accordance with the Cumulant model. The profiles are related with four microemulsions emphasized in the optimization process, where MELSA 8 was considered the optimal system.

**Figure 7:**
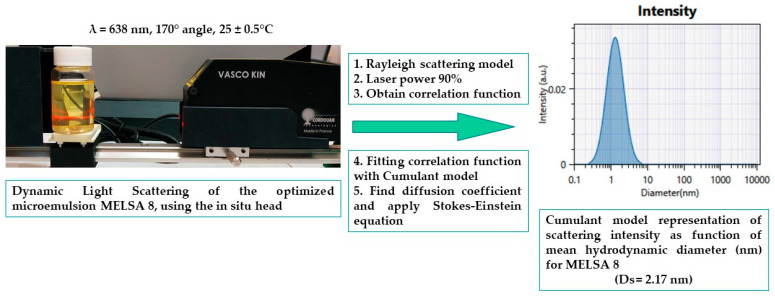
Representation of dynamic light scattering for the optimized microemulsion MELSA 8, using the in-situ head of ViscoKin particle analyzer, and five steps that are followed to assess the response related with the Cumulant algorithm.

Figure 7 visually represents a complete course of the experimental process of dynamic light scattering for MELSA 8 microemulsion, beginning with laser scattering analysis, followed by data acquisition and depiction of mean droplet size, associated with the Cumulant model profile expressed as scattering intensity as a function of droplet size (nm).

By appealing to the clarity parameter, from a qualitative point of view, and associating droplet size values, it can be observed that clarity better corresponds with systems characterized by low Ds values.

In the same way, DLS determinations offered particular information about the homogeneity of the systems and distribution width. The Polydispersity index for the tested microemulsions varied between 0.039 ± 0.010 and 0.557 ± 0.038, describing two types of dispersions: homogeneous systems and polydisperse microemulsions, which can be better evaluated using distribution plots related with an SBL algorithm. The values of PDI were placed between 0–1 domain required for colloidal nanodispersions. It was remarked that homogeneity was specifically found for MELSA 2, MELSA 4 and MELSA 6 microemulsions prepared with higher levels of Tween 80/PG, and for only one microemulsion stabilized with 20% Tween 80/PG, namely MELSA 3. For these systems, the polydispersity index varied between 0.039 and 0.119. From this group, MELSA 6 which was studied as one of the optimal, showed a monomodal distribution with a single maximum peak at 1.49 nm. Far beyond this threshold, it was considered that polydispersity was peculiar for microemulsions based on medium and low levels of Tween 80/PG. Even if MELSA 8 had a maximum amount of Tween 80/PG, selection of a maximum level of oil phase of 2% determined a bimodal distribution with one peak of high intensity assessed at 1.68 nm and another one of reduced intensity at 5.58 nm. In the same manner, the optimized MELSA 11 and MELSA 12 systems were characterized by trimodal and bimodal distributions. For MELSA 11, three peaks were observed: the first was of high intensity and tight at 0.7 nm, followed by two larger ones: the second was observed at 2.83 nm, and the third at 24.02 nm. In the last case, distribution of particles was characterized by two peaks: the first of low intensity at 1.05 nm, and the second of high intensity at 28.81 nm (data are not presented). From a statistical point of view, no relationship was found between PDI variation and composition of microemulsions (*p* > 0.05).

Considering zeta potential as an important parameter for microemulsions stabilization, particles were discovered with a low negative electric charge. Traditionally, values of zeta potential around +/− 30 mV are attributed for systems with a high stability, defining electrostatic repulsions that occur between particles, as opposed to aggregative trends related to creaming or Ostwald ripening instability phenomena. However, microemulsions stabilized with non-ionic surfactants, in our case Tween 80, associated with low levels of lecithin, zeta potential had the tendency to be closer to a zero value. In this way, zeta potential was placed between −3.83 ± 0.01 mV and −1.38 ± 0.05 mV, signifying an apparent pattern of instability, which cannot be attributed to these systems. The lack of electrostatic repulsions is known to be replaced by other stabilization forces, such as steric repulsions and dispersion forces.

### 3.7. Superficial Analysis

To our knowledge considering literature reports, goniometric study has rarely been proposed in the research of microemulsions. However, the study of superficial properties must be considered of major significance when we make reference to superficial phenomena of microemulsions in contact with solid surfaces, as well as their wettability character, and depiction of work of adhesion. Understanding the problematic of these parameters can serve to create a guideline in order to explain the behavior of colloidal systems in topical application, as in our case, or when in contact with other biomembranes. In this context, a good spreadability of a topical product can be obtained with low adhesion, involving contact angles which describe a better wettability. In the same manner, the impact of formulation parameters can be studied in adhesion/cohesion phenomena and their physical interactions with solid surfaces. Hereinafter, our experimental approach was based on the determination of three main parameters, specifically: superficial tension at liquid/gas interface (γ_LG_), contact angle (CA), and work of adhesion (W), which can be visualized in Table 5. Their quantification was performed by applying a Young equation, which was fitted with two models of study: the pendant drop model and the contact angle model.

**Figure 8:**
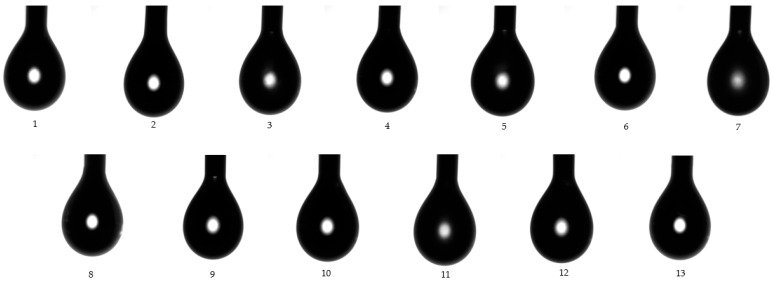
Captures for microemulsion drops (1–13) in pendant drop model study, evaluating γ_LG_ at 25 ± 0.5 °C.

In this order, the superficial tension was determined for each microemulsion using both models of study. According to the first model, superficial tension of drops from each microemulsion sample was determined at the liquid/gas interface (γ_LG_), before the drops fell on the glass slide under gravitational force. Figure 8 visually represents images of each drop from MELSA 1–MELSA 13 systems, which were released from a Hamilton syringe and subjected to measurements. Superficial tension varied between 32.57 ± 0.34 mN/m and 37.36 ± 0.16 mN/m and was significantly influenced by Tween 80/PG (%) (*p <* 0.05). The minimum value corresponded to MELSA 2, while the maximum value was depicted for MELSA 5. It can be observed that the higher values of superficial tension were specific for microemulsions with Tween 80/PG of 20%, and consequently higher concentrations of water. Related results were obtained in the group of systems with the medium level of Tween 80/PG of 30%. The differences between the two groups regarding the superficial tension values were not significant. Considering the last group, the influence of Tween 80/PG at the maximum level of 40% had a high persistence among γ_LG_ values. The impact of lecithin as a second surfactant was quite significant, the values being placed between 32.57 ± 0.34–33.73 ± 0.24 mN/m.

The study of the contact angle offered a rigorous perspective and helped us to understand, explain and propose associations between superficial tension, contact angle, and their relationship with work of adhesion. In Table 6, parameters expressed as mean values with their standard deviations are shown. Following this model, the drop released from the Hamilton syringe was captured when it adhered to the solid surface in 0.064 s. The values of the superficial tension were found to be statistically different (*p* = 0.0065), compared to those obtained in the first model. Both groups were analyzed as dispersions by applying the ANOVA single factor test. Short descriptive statistics and the results of the test are presented in Table 6.

In the concept of contact angle model, to this level, γ_LG_ represents the result of the forces that will oppose a complete wetting phenomenon, determining interactions between adhesion and cohesion forces, and formation of various angles between 40.74 ± 0.70–57.91 ± 0.38°, which describe a moderate wettability. Complementary to this, the values of γ_LG_ varied between 33.38 ± 0.46–52.90 ± 1.82 mN/m, promoting a variation in the work of adhesion on a larger domain of 52.72 ± 1.44–81.00 ± 2.49 mN/m. The values of γ_LG_ were significantly influenced by Tween 80/PG (%) and the oat oil (%) (*p <* 0.05). Lecithin (%) offered a supplementary significant effect on the work of adhesion (*p <* 0.05). Thereby, for microemulsions with a minimum level of Tween 80/PG of 20%, modification in lecithin concentration slightly influenced the values of γ_LG_. In the case of MELSA 1 and MELSA 3, the elevation of γ_LG_ with 1 mN/m produced an insignificant increase in contact angle, and consequently in the work of adhesion. Decreasing the level of lecithin to 0.3% in the case of MELSA 7, and adding 2% oil phase, will determine the action of a low γ_LG_, and formation of a contact angle of 43.27°, promoting a proper adhesion defined by a work of adhesion of 58.64 mN/m. On the other hand, in the case of MELSA 5, the hydrophilic effect of water at the maximum amount of 77.2%, influenced γ_LG_ to reach 41.31 mN/m. The forces implied in the mechanical work were powerful, so the work of adhesion had a higher value, determining a display of drops with the smallest medium angle of 40.73°. From this group, a good work of adhesion was obtained for MELSA 7 with Tween 80/PG 20%, lecithin 0.3%, and oat oil 2%.

Increasing Tween 80/PG concentration at 30%, it was observed another profile for contact angles in domain of 46.75 ± 4.14–54.59 ± 1.86°, which were formed with a moderate mechanical work that supported adhesion. Beginning from MELSA 9, its composition with lecithin 0.1% and oil phase 1%, determined detection of γ_LG_ of 37.63 mN/m, promotion of an angle of 51.93°, and a medium work of adhesion of 60.80 mN/m. This profile can be compared with that of MELSA 3. The increase in viscosity was favorable for the angle elevation, and consequently for the work of adhesion. Hereinafter, in MELSA 10 case, γ_LG_ increased proportionally with the use of the maximum level of lecithin and influences the formation of drops of 54.04° and the highest work of adhesion from this group. In this case, cohesion forces opposed to the wettability of the surface. Following the case of MELSA 11, the adhesion was more favorable, even if the angle was comparable with the anterior one. With a γ_LG_ of 33.38 mN/m, work of adhesion was proper at a minimum level of lecithin, and a maximum level of oil. Maintaining oat oil concentration at constant level, and increasing lecithin at 0.5%, γ_LG_ facilitated a displaying of drops with a smaller angle, but under the effect of higher forces implied in adhesion. Similarly, it can be observed in the last case, for MELSA 13. From this group, a better adhesion was obtained for MELSA 11 with Tween 80/PG 30%, lecithin 0.1%, and oat oil 2%. To conclude this series, a short brief point considering the influence of lecithin and oil phases on the dynamic of microemulsions at the contact with a surface can be made. By reference to the four microemulsions (MELSA 9, MELSA 10, MELSA 11, and MELSA12) the following were emphasized:

- Between MELSA 9 and MELSA 10, the variation of lecithin through a higher concentration, determined an increase in the angle and the work of adhesion.
- Between MELSA 9 and MELSA 11, the variation of oil through a higher concentration, determined an increase in angle, but a decrease in the work of adhesion. In this case, a wettability phenomenon is expected to occur.
- Between MELSA 11 and MELSA 12, the variation of lecithin, keeping constant the maximum amount of oil, determined an increase in the work of adhesion, but a decrease in the contact angle. The oil phase improved the wettability of the surface.
- Between MELSA 10 and MELSA 12, the variation of oil, keeping constant the maximum amount of lecithin, determined a decrease in the contact angle, and a decrease in the work of adhesion. The oil phase improved the wettability at the level of the surface.

Finally, the adjustment of the Tween 80/PG concentration at 40% significantly influenced the properties of adhesion of microemulsions. Elevation of lecithin concentration from 0.1% to 0.5% in the case of MELSA 2 and MELSA 4, produced an increase in γ_LG_. Consequently, the work of adhesion increased from 67.52 mN/m up to 76.91 mN/m, without modification in the contact angle value. As with the previously discussed series, lecithin had an additional impact on the induction of a higher work of adhesion, limiting the wettability of surface. In the case of MELSA 6 and MELSA 8, an increase in the contact angle can be observed. The maximum value of 57.91° was reached, with a work of adhesion of 81 mN/m at a minimum level of oil of 1%, and lecithin 0.3%. At a maximum level of oil of 2%, the drop adhered much better to the surface, keeping a similar angle. Weaker mechanical forces contributed to a good wettability in the last case. It can be seen that a better adhesion was obtained for MELSA 8 with Tween 80/PG 40%, lecithin 0.3%, and oat oil 2%.

**Figure 9:**
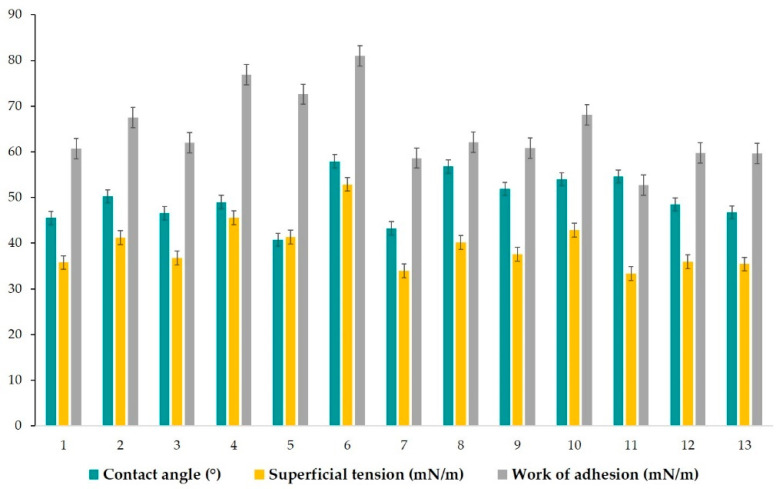
Representation of contact angle, superficial tension and work of adhesion and their variation, for each microemulsion (1–13) in a comparative manner, after the contact angle model study.

In accordance with those data, Figure 9 shows the variation of the mean contact angle, superficial tension, and the work of adhesion for each microemulsion in a comparative way.

**Figure 10:**
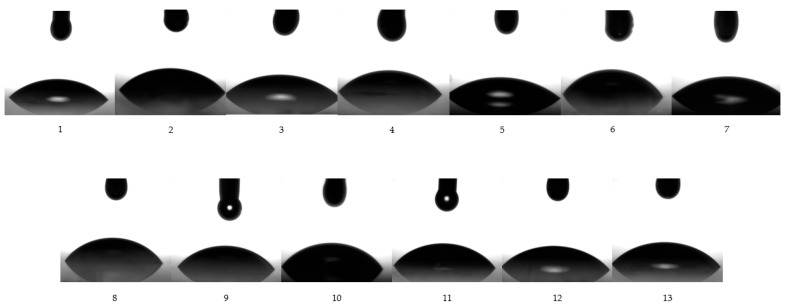
Captures for microemulsion drops (1–13), placed on the glass slide, in contact angle model study, evaluating γ_LG_ and CA at 25 ± 0.5 °C.

In agreement with the data previously discussed, Figure 10 presents captures for each drop of microemulsion sample (noted 1–13), studied using the contact angle model. Their behavior can be observed in the matter of wettability and adhesion in contact with a solid surface.

### 3.8. Optimization of Critical Quality Attributes for Microemulsions Using Box–Behnken Design

The optimization process of critical quality attributes of microemulsions consisted in the development of a 3^3^ fractional factorial design characterized by 13 experimental runs in the Minitab software. The analysis of responses namely Y_1_: viscosity (cP), Y_2_: mean droplet size (MPS)—Ds (nm), Y_3_: work of adhesion—W (mN/m), as a function of formulation factors defined as X_1_: Tween 80/PG (%), X_2_: lecithin (%), and X_3_: oat oil (%), was conducted by applying the response surface methodology (RSM). Accordingly, the following were considered as valuable for this stage: the response surface regression accompanied by a statistical interpretation for each response, and the highlights of contour plots and response surfaces. Subsequently, Pareto charts were considered representative plans that can emphasize the significance of independent variables, and interactions that can influence the response. Associated with the experimental responses, predicted values were determined based on the fitted model of the second order polynomial equations.

The expected optimized system must comply with a series of criteria: a high grade of viscosity which is well-known as a required parameter to attain a good consistency and spreadability on skin tissue; a low mean droplet size obtained with a maximum level of oil phase, and a low work of adhesion which can sustain the spreadability and wettability at skin surface level, sustaining in the same manner the initiation of penetration processes, and the bioavailability of active substances in the targeted affected layers of the skin.

Table 7 shows independent and dependent variables analyzed using design of experiment steps for 13 formulations equivalent to 13 runs performed in the software using the Box–Behnken design model. Separately, a clarity parameter was considered a qualitative mark, applied as an exclusion criterion, to find the optimum level in the microemulsion formulation. Thus, systems with good clarity graded as “+++” will be considered in the optimization in the first instance.

#### 3.8.1. Optimization of Viscosity

Optimization of viscosity was proposed to find the microemulsion-based systems characterized by good spreadability at skin level. It is well known that the flow behavior of topical formulations will influence the dynamic of the product upon administration. On this side, extrusion from the recipient, the spreading on tissue, and the diffusion process through the deeper layers of skin will be mostly influenced by the viscosity and by default, the main formulation factors that modulate it.

In accordance with the results obtained in the response surface regression analysis, the viscosity parameter was significantly influenced by the Tween 80/PG concentration (X_1_) (*p <* 0.05). The second order polynomial equation (Equation (6)) that fitted the response (Y_1_) was proposed hereinafter:

(6) $$Y_{1}=304\;-\;{33.8X}_{1}{+386X}_{2}{+63X}_{3\;}+0.899X_{1\;}^{2}{-\;84X}_{2\;}^{2}+3.9X_{3\;}^{2}-\;13.97X_{1}X_{2\;}-\;3.13X_{1}X_{3\;}{+4X}_{2}X_{3}$$

The values of viscosity (cP) varied in the domain of 16.94–292.69 cP. At the maximum level of Tween 80/PG (X_1_), a group with the highest levels of 188.17–292.69 cP was outlined, determining a decrease in fluidity and a better stability. The variation of X_2_, and X_3_ was not of significance in this case (*p* > 0.05), as well as their squares and interactions. To exemplify this, ANOVA analysis results are shown in Table 8. Only one square defined as $X_{1\;}^{2}$ was of significance, having a positive effect for the Y_3_ response.

**Figure 11:**
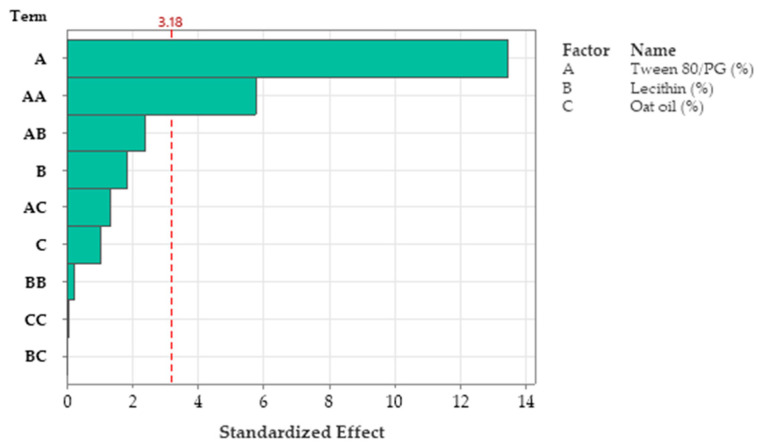
Pareto chart of standardized effects for viscosity response, emphasizing main factors implied in modulation of response, namely A: Tween 80/PG, and AA: Tween 80/PG × Tween 80/PG as squared term, where the factors AB: Tween 80/PG × Lecithin, B: Lecithin, AC: Tween 80/PG × Oat oil, C: Oat oil, BB: Lecithin × Lecithin, CC: Oat oil × Oat oil, and BC: Lecithin × Oat oil were insignificant.

R-squared obtained for the model was 98.76%, while the adjusted one by reference to degrees of freedom was 95.03%. The analyzed model was considered significant (*p <* 0.05). The Pareto chart presented in Figure 11 presents the standardized effect of terms explained by the regression equation. Thus, the stabilizer mixture formed with Tween 80/PG (2:1) had the main effects on viscosity, as we previously emphasized. The narrowed domains of concentration for lecithin as a second surfactant, and for oat oil were not representative for viscosity variation.

**Figure 12:**
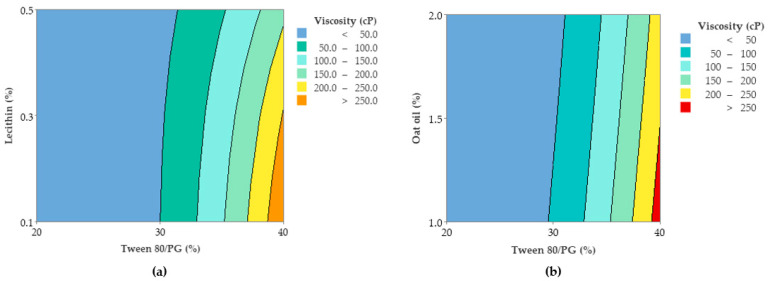
Contour plots representing viscosity response (Y_1_) as function of (**a**) Tween 80/PG (%), Lecithin (%), and (**b**) Tween 80 (%), Oat oil (%).

**Figure 13:**
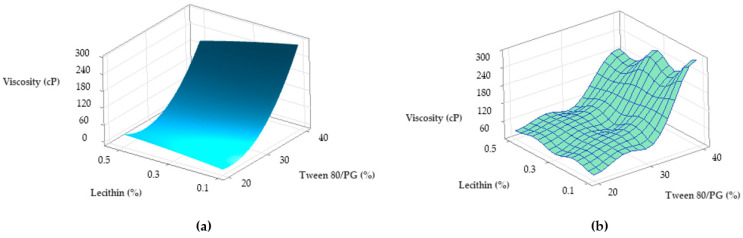
Surface plots representing viscosity response (Y_1_) as a function of Tween 80/PG (%) and Lecithin (%): (**a**) Surface representation in gradient color, and (**b**) Wireframe model.

In accordance with these findings, contour plots and surface plots for Y_1_ response were projected, which are shown in Figure 12 and Figure 13.

Considering Figure 12, a contour plot—case (a)—representing viscosity (cP) as a function of X_1_ and X_2_ factors was considered representative to explain viscosity variation in low, medium, and high levels of Tween 80/PG (%). It can be observed that the response is specifically sensitive for X_1_ variation. By reference to Y axis, flat lines signified the negligible effect of lecithin for Y_1_ response.

Similar observations are suggested by the contour plot of viscosity (cP) as a function of X_1_ and X_3_ factors in the case (b). The increase of Tween 80/PG (%) from 20% up to 40%, determined the elevation of viscosity, attaining the maximum value over 250 cP, but in an independent manner. That is why the flat lines target the X axis, without determining a significant effect for the Y axis variable.

The surface plots shown in Figure 13 were designed to highlight a tridimensional fit for viscosity response. The case attributed to Y_1_ variation as a function of Tween 80/PG (%) and lecithin (%) was chosen and projected as a surface in gradient color—case (a), and wireframe plot—case (b). In the first case, a proportional elevation of viscosity with the increase of Tween 80/PG (%) is observed from a lighter blue tone through to a darker shade at any level of lecithin. In addition, the embossed surface emphasizes the maximum points which were related with the optimal expected results. Thereby, it can be appreciated that valuable viscosity responses which can be relevant for a therapeutic purpose regarding parameters such as—clarity, spreadability, stability—were obtained at the highest level of Tween 80/PG of 40%. The systems were MELSA 2, MELSA 4, MELSA 6, and MELSA 8.

**Figure 14:**
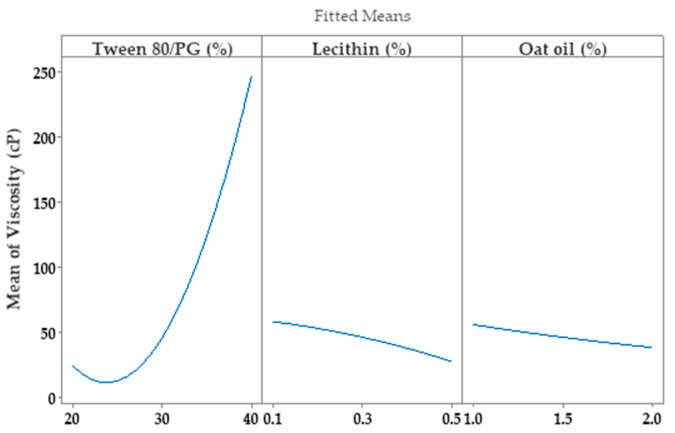
Main effects plot for viscosity response (Y_1_), considering individual effect of each independent variable.

Consequently, the optimization study on the viscosity parameter (Y_1_) was completed after representation of factorial plots and prediction of optimal viscosity, taking into consideration the aforementioned criteria. On this path, the main effects plot for viscosity, shown in Figure 14, represents the final evidence whereby the Tween 80/PG (%) factor critically influenced viscosity, while the rest of the terms (lecithin (%) and oat oil (%)) had a non-significant effect on viscosity. Keeping X_1_ as the main factor, in the interval 20–30% a lower effect on viscosity was observed, with a minor decrease, followed by an elevation, attaining a significant effect of over 30%.

As a final point, a predictive response on optimal viscosity was proposed. The targeted goals to successfully run the response optimizer consisted in maximizing viscosity and imposing constraints on X_3_ variable, by setting a high level of 2% as desired. The obtained solution indicated a system with X_1_: 40%, X_2_: 0.1%, and X_3_: 2% as the best fit to obtain a high response. Predicted viscosity was 263.83 cP, and the composite desirability was 0.8953. The solution of predictive response is presented in Table 9.

**Figure 15:**
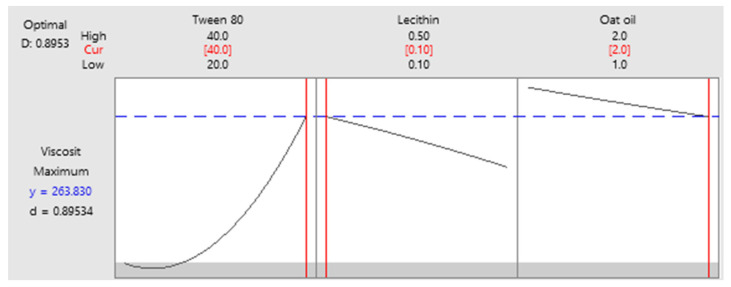
Predictive optimization plot for viscosity response as function of three setting parameters: X_1_—maximized, X_2_—no constraints, and X_3_—maximized, with a composite desirability of 0.8953.

The result can be corroborated with the data obtained in the experimental process, emphasizing two systems that are characterized by high viscosities around predicted values, namely MELSA 6 with a viscosity of 287.77 cP, and MELSA 8, with a viscosity value of 217.73 cP. Considering the criterion of oil phase selection at the maximum level of 2%, it can be seen that MELSA 8 is eligible as an optimized model system and close to the predicted one. Predictive optimization was defined using the optimization plot which describes the optimal settings to assess the response in relationship with the variables main effects and is presented in Figure 15. The composite desirability value was close to a value of 1, which meant that the settings of the model were selected to assure a favorable response near ideal system.

#### 3.8.2. Optimization of Mean Droplet Size (Ds)

The mean droplet size (Ds) of MELSA 1–MELSA 13 microemulsions was optimized with the aim of obtaining a model system characterized by a low hydrodynamic diameter, under 10 nm. It can also be emphasized that the formation of small particles as lipophilic nanocores which incorporated salicylic acid 0.5% and were dispersed in a hydrophilic medium of a hyaluronate based aqueous solution, under the stabilization effect of Tween 80/PG and the lecithin mixture. The main target in this case was the optimization of a clear and stable system containing the maximum amount of oil at 2%.

Regarding the response surface regression analysis, Ds was significantly influenced by Tween 80/PG (%)—X_1_ term, oat oil (%)—X_3_ term, and the two-way interaction between the two terms: X_1_X_3_ (*p <* 0.05). Thus, the quadratic polynomial equation (Equation (7)) that fitted the response (Y_2_) is presented below:

(7) $$Y_{2}=7.5\;-0.04X_{1}\;-\;67.4X_{2}+6.8X_{3\;}+0.0350X_{1\;}^{2}+75.0X_{2\;}^{2}+14.5X_{3\;}^{2}\;-\;1.00X_{1}X_{2\;}-\;1.685X_{1}X_{3\;}+48.5X_{2}X_{3}$$

The mean droplet size values varied between 1.58 nm and 37.72 nm and were highly influenced by Tween 80/PG and oat oil levels. The highest values of Ds were attributed to systems with low levels of Tween 80/PG, medium values of Ds were specific for systems with medium levels of Tween 80/PG, while small Ds results were associated with the maximum amount of the same mixture. Subsequently, oat oil had a particular influence on Ds, modulating particle dynamics and the stability of the projected systems. By reference to regression equation, it can be observed that the X_1_ term had a negative effect on Ds in the sense of minimization. By contrast, the X_3_ term had a positive coefficient, including a positive effect on Ds elevation. Considering the interaction of the two variables, the X_1_X_3_ term had a negative coefficient, associated with a negative effect directed through a minimization of Ds as a desirable consequence.

Table 10 presents the data extracted in the ANOVA analysis, with significant and insignificant terms of regression equation, as a function of the *p* value, at a confidence level of 95%.

**Figure 16:**
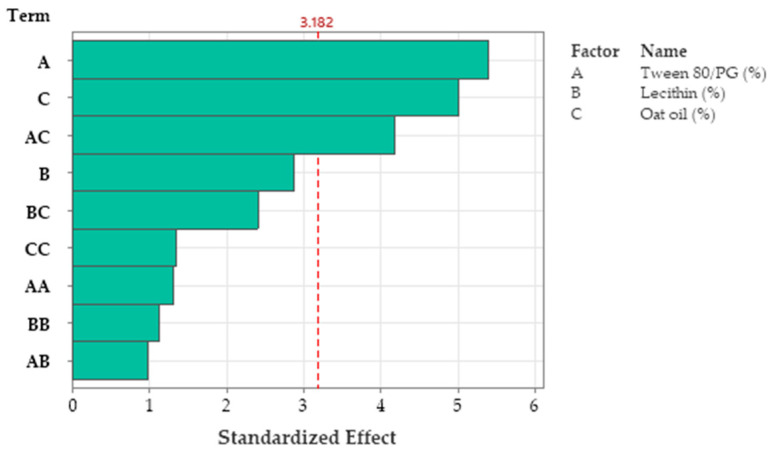
Pareto Chart of standardized effects for Ds response, emphasizing main factors implied in the definition of response, namely A: Tween 80/PG (%), C: Oat oil (%), and AC: Tween 80/PG (%) × Oat oil (%) as interaction term, where the factors B: Lecithin, BC: Lecithin × Oat oil, CC: Oat oil × Oat oil, AA: Tween 80/PG × Tween 80/PG, BB: Lecithin × Lecithin, and AB: Tween 80/PG × Lecithin were insignificant.

R-squared obtained for the model was 96.76%, while R-squared for the adjusted one by reference to degrees of freedom was 87.04%. The analyzed model was considered significant (*p <* 0.05). Figure 16 presents the standardized effect of terms explained by a fitted quadratic equation, using a Pareto chart. In this case, the main effect on Ds was attributed to the stabilizer mixture of Tween 80/PG (term A), followed by oat oil level (term C), and their interaction (term AC). The lecithin effect and other terms derived from it were not significant for the model.

**Figure 17:**
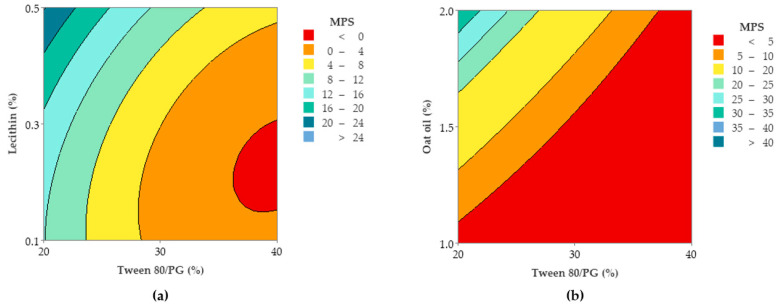
Contour plots representing Ds response (Y_2_) variation as function of: (**a**) Tween 80/PG (%), Lecithin (%), and (**b**) Tween 80/PG (%), Oat oil (%).

**Figure 18:**
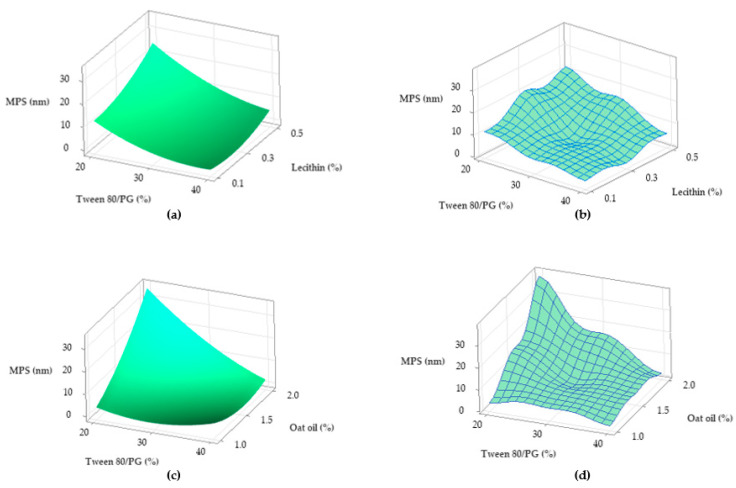
Surface plots representing: Ds response (Y_2_) as a function of Tween 80/PG (%) and lecithin (%) in two cases—(**a**) Surface representation in gradient color; (**b**) Wireframe model, and Ds response (Y_2_) as function of Tween 80/PG (%) and Oat oil (%) in two cases—(**c**) Surface representation in gradient color; (**d**) Wireframe model.

To observe the response variation as a function of the independent variables, the contour plots and surface plots were designed and presented in Figure 17 and Figure 18.

Focusing on the contour plots for the Y_2_ response presented in Figure 17, the two representative cases that explain particle dispersion in the domain of 1–40 nm, taking into consideration the significance of formulation variables, can be observed. In the first representation—case (a), the response is shown in gradient color signifying the variation of Ds from high dimension particles (dark blue color), through to small particles (red and orange colors), as a function of X_1_ factor and X_2_ factor. The response is specifically sensitive to the X_1_ variation, and it can be deduced that this is due to the presence of curvature lines directed through the X axis specific to the Tween 80/PG variation. Additionally of note is the narrowing of an extremely small particle domain, associated with the selection of Tween 80/PG at the maximum level of 40%. A wider domain of particles around 4–8 nm was marked at the medium level of Tween 80/PG of 30%. An increase in droplet size was determined by decreasing in Tween 80/PG level to the inferior limit. On the other hand, the effect of lecithin was not significant for the response. Particles in the fixed domain can be formed either at low or high levels of lecithin.

In the second representation—case (b), Ds response is presented in gradient color, emphasizing its variation from particles with higher diameter (green color), through to small particles (red color), depending on X_1_ factor and X_3_ factor. Assuming the aforementioned considerations concerning X_1_X_3_ interactions deduced from the regression equation, the Ds response is specifically sensitive to both variables. The highest sensitivity is attributed to the X_3_ variable, and the contour lines oriented through the Y axis can be observed. Therefore, a red domain is specific for particles with a diameter under 5 nm, which can be formed when X_1_ is settled to a low level and associated with a low level of X_3_. In the same manner, at a maximum level of X_1_, small particles were formed when X_3_ was settled at any of the three levels of variation. Finally, setting up the X_1_ and X_3_ parameters at a medium level is favorable to attain small particles. An increase of X_3_ to the maximum level, using medium levels of X_1_, will sustain the obtaining of particles in a larger domain of up to 40 nm. To conclude, concentrations of Tween 80/PG of 40% are required in order to attain small particles, if the oil phase is considered to be 2%, as well as in the case when the system contains a medium concentration of stabilizer mixture of 30%, prepared with small, medium, or a maximum level of oil phase.

From a tridimensional perspective, Figure 18 emphasizes the response surfaces for Ds in accordance with both cases previously exemplified. In the first case related with the Y_2_ variation as a function of Tween 80/PG (%) and lecithin (%), a surface in gradient color—case (a), and a wireframe plot—case (b) was projected. On the surface plot can be seen a 3D variation of Ds as function of Tween 80/PG (%), in the sense of minimization from a lighter green shade to a darker tone on the X axis direction, without significant influence of the lecithin level. Moreover, the wireframe model interprets these effects due to the embossed view presented with maximum and minimum points.

In the second case related with Y_2_ variation as a function of Tween 80/PG (%) and oat oil (%), a surface projected in gradient color—case (c), and the associated wireframe plot—case (d) can be seen. The surface plot shows the variation of Ds in the sense of minimization from a lighter blue color to a darker green tone with respect to the oil phase amount. The area of small Ds is attributed to the use of medium to high levels of Tween 80/PG at any concentration of oil phase. In addition, the points of maximum and minimum targeting of the Ds response are shown on the wireframe in case (d).

**Figure 19:**
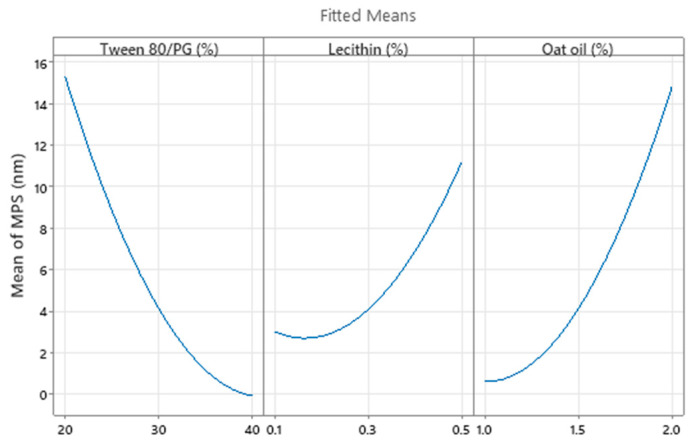
Main effects plot for Ds response (Y_2_), considering individual effect of each independent variable.

The optimization study on the Ds parameter (Y_2_) required the study of variables’ main effects using factorial plots, the prediction of an optimal system, and its corroboration with experimental results. Thus, the main effects plot for Ds presented in Figure 19 represents a cumulative analysis which proves the impact of Tween 80/PG and oat oil factors as critical attributes for the droplet size of microemulsions, as earlier explained. These two factors are graphically presented in an opposite manner. A decrease in droplet size through the expected domain can be described, along with the increase in Tween 80/PG level and the minimization of oil level. Lecithin exerted a lesser effect on Ds with an isolated response observed at 0.5% in the direction of particle enlargement, but cannot be considered significant for the model.

Based on these main effects discovered using graphical methods, a predictive response on optimal Ds was studied and compared with possible real solutions. Optimization parameters were set in order to minimize Ds to a targeted value, between 1–10 nm, namely 5 nm. Constraints were imposed only on oat oil as a maximized value, keeping Tween 80/PG in the domain of 30–40%. The fitted solution determined the following levels for each factor: X_1_: 35%, X_2_; 0.2214%, X_3_: 2%, with a composite desirability of 1.0000. The solution of predicted response is shown in Table 11.

**Figure 20:**
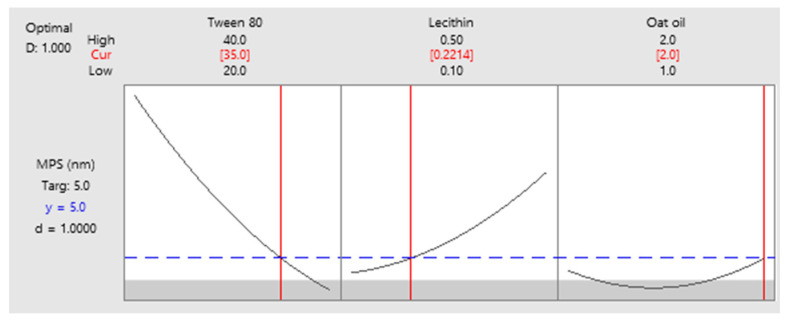
Predictive optimization plot for Ds response as function of three setting parameters: X_1_—kept between 30–40%, X_2_—without constraints, X_3_—maximized, with a composite desirability of 1.000.

By reference to the experimental results, it can be stated that MELSA 8 and MELSA 11 are two systems that are characterized by a mean droplet size of 2.17 nm and 6.45 nm, respectively. The systems are considered to fulfill the requirements in matter of composition. MELSA 8 had the highest level of Tween 80/PG and a medium level of lecithin (0.3%), closer to 0.22%, while MELSA 11 had a medium level of Tween 80/PG and a lower level of lecithin (0.1%), closer to 0.22%. By applying the exclusion criterion based on clarity observed over the period of one month, MELSA 8 is considered the reference system. The predictive optimization session is graphically described in Figure 20. The Ds response is marked by a blue line for each factor, and it was perpendicularly crosslinked with the red line which signifies the optimized level of the parameter. In each point of the three intersections, a grey line passes in order to produce the desired effect, in accordance with the main effects drawn in the previous plot.

#### 3.8.3. Optimization of Work of Adhesion 

Work of adhesion represents an important parameter that can explain the behavior of a topical formulation at the application site. The adhesion effect was studied as an excellent fit between superficial properties and wettability characteristics of microemulsions. The tested microemulsions exhibited good wettability attributes quantified by contact angles between 43.27–57.91°, and the work of adhesion values were placed in a larger domain of 52.7–81.00 mN/m, which were correlated with their composition.

Optimization of work of adhesion was performed in order to find a system characterized by adequate adhesion properties due to the adhesion implication on spreadability and interaction of the formulation ingredients with a solid surface, such as skin level.

In the case of work of adhesion as a dependent variable, the response surface regression evaluation revealed the quantification of Y_3_ using a reduced second order polynomial equation, as a cause of the lack of significance for the model sustained by a full quadratic equation (*p* > 0.05). In this way, the regression equation (Equation (8)) is presented as follows:

(8) $$Y_{3}=140.2\;-\;4.20X_{1}+23.8X_{2\;}-\;27.5X_{3\;}+0.0769X_{1\;}^{2}-\;13.5X_{2\;}^{2}+5.05X_{3\;}^{2}$$

As a result, work of adhesion (Y_3_) was significantly influenced by four terms presented in the regression equation, in particular: Tween 80/PG (%)—X_1_ term, lecithin (%)—X_2_ term, oat oil (%)—X_3_ term, and a squared term $X_{1\;}^{2}$, with *p <* 0.05 according to ANOVA analysis. It can be appreciated that Tween 80/PG (%) had a negative effect on the work of adhesion, along with oat oil (%), in the direction of response minimization. On the other hand, lecithin (%) was considered favorable to increase the response, along with the squared term attributed to Tween 80/PG (%).

In Table 12, the data calculated in ANOVA analysis, with significant and insignificant terms presented in the second order polynomial equation as a function of the *p* value, at a confidence level of 95% can be visualized. The R-squared obtained for the fitted model was 90.98%, while the adjusted R-squared by reference to degrees of freedom was 81.95%. In conclusion, the model was considered significant at *p <* 0.05.

**Figure 21:**
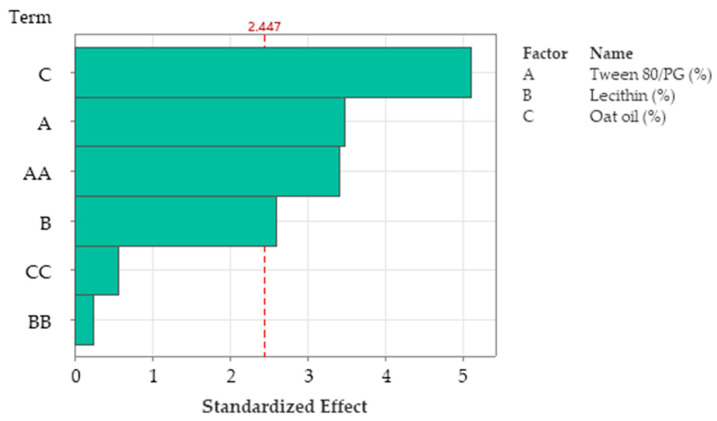
Pareto Chart of standardized effect for W response, emphasizing the main factors implied in the definition of response, namely C: Oat oil (%), A: Tween 80/PG (%), AA: Tween 80/PG (%) × Tween 80/PG (%) as squared term, and B: Lecithin (%), where the factors CC: Oat oil × Oat oil and BB: Lecithin × Lecithin were insignificant.

In what follows, the standardized effect for the Y_3_ response is further presented in Figure 21 as a Pareto chart, taking into consideration the main terms of impact for the work of adhesion of microemulsions to a solid surface. Thus, the main effect on the work of adhesion was considered to be produced by oat oil, followed by the stabilizer mixture of Tween 80/PG and its squared term, and lecithin as a lipophilic surfactant. The other squared terms implied in the model were insignificant.

**Figure 22:**
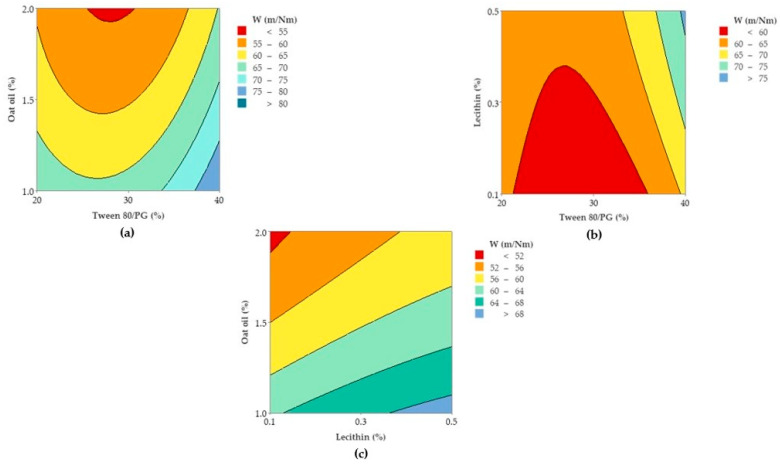
Contour plots representing W response (Y_3_) variation as a function of: (**a**) Tween 80/PG (%), Oat oil (%), (**b**) Tween 80/PG (%), Lecithin (%), and (**c**) Oat oil (%), Lecithin (%).

**Figure 23:**
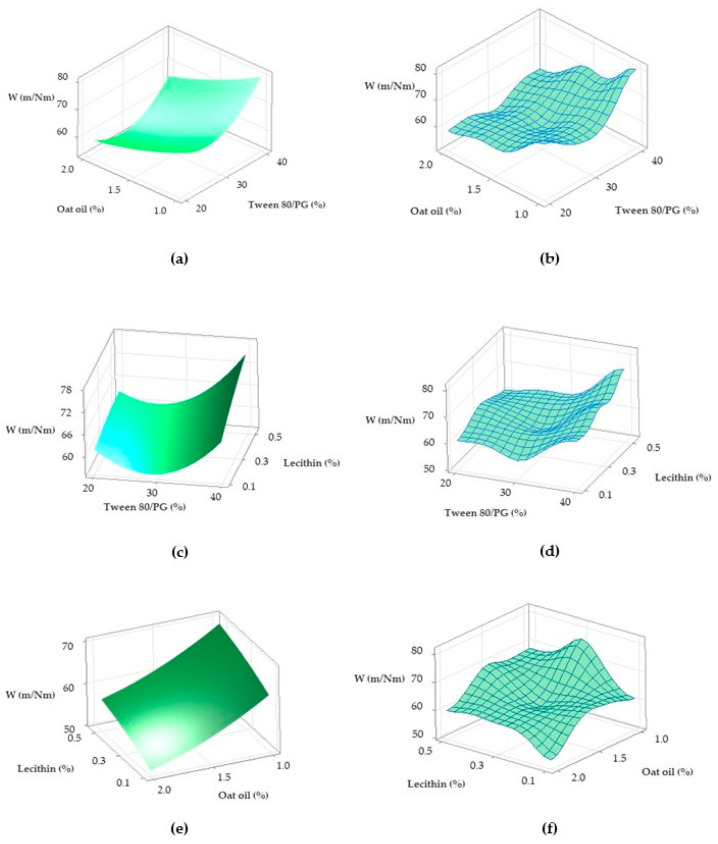
Surface plots representing: W response (Y_3_) as a function of Tween 80/PG (%) and Oat oil (%), in two cases—(**a**) Surface representation in gradient color, (**b**) Wireframe model; W response (Y_3_) as function of Tween 80/PG (%) and Lecithin (%), in two cases—(**c**) Surface representation in gradient color, (**d**) Wireframe model; W response (Y_3_) as function of Oat oil (%) and Lecithin (%) in two cases—(**e**) Surface representation in gradient color, and (**f**) Wireframe model.

Targeting the main effects that describe the adhesion phenomena of microemulsions studied in the goniometric analysis, contour plots, and surface plots were projected and are presented in Figure 22 and Figure 23.

Contour plots for the Y_3_ response are presented in Figure 22 and bring out the main effects of the three formulation factors, highlighting three cases of interdependence, denoted (a), (b), and (c).

In the first case (a), a contour plot that defined work of adhesion response as a function of X_1_ and X_3_ factors can be observed. It is important to mention that the X_3_ factor namely oat oil (%) prevails as the main ingredient in the modulation of adhesion. Both factors had a significant sensitivity for response assessment, being described by well-defined curved lines that were projected on the Y axis and the X axis. Following the gradient colors exposed below, it can be seen that at high levels of Tween 80/PG and low levels of oat oil, the work of adhesion had a maximum value of 81.00 mN/m. By increasing oil concentration, the work of adhesion slightly decreased to over 70 mN/m, then reaching over 60 mN at the maximum level of oil. In a similar fashion, at medium and minimum levels of Tween 80/PG and lower oil levels, the work of adhesion remained in the middle green area, around 65 mN/m. The increase in the oil phase level involved a decrease in the work of adhesion, described by extended yellow and orange-like patterns. The minimum W value was attributed to a small red domain, associated with the selection of Tween 80/PG (%) at medium level, and the oil phase at maximum level.

In the second graphic (b), the contour plot defined the response as a function of the X_1_ and the X_2_ factor. In this situation, lecithin exerted a small effect on W, being considered a factor that was implied in the minimization of response on the entire domain of Tween 80/PG variation. This fact is justified by the presence of larger red and orange like areas related to W responses under 65 mN/m. The narrowed domain of response over 65 mN/m was determined by the X_1_ factor. Hence, the contour lines are directed through abscissa, increasing the sensitivity response for Tween 80/PG.

To succeed in final observations, the last case (c) presents a contour plot for Y_3_ response as a function of the X_2_ and X_3_ terms. Observed on the plot are a different allure of contour lines which are directed onto the ordinate, and to a lesser extent, through the abscissa, which is related to the sensitivity of the oil phase effect on the work of adhesion. The area related to a high response of over 60 mN/m is represented by the two green tone domains for systems with low or medium levels of oil associated with low or medium amounts of lecithin. An isolated case of high W over 65 mN/m is depicted on the blue area and is specific to the maximum level of lecithin and low oil concentrations. By contrast, variation in the work of adhesion in the opposite sense is associated with the increase of oil content, but independent of lecithin.

Powerful evidence for the presented cases is further emphasized by adding to the discussion the effect of variables upon the response from a tridimensional point of view, as can be visualized in Figure 23, as surface representations and wireframe models for the three main cases.

Firstly, surface plots were designed in order to visualize the Y_3_ response in a 3D manner as a function of Tween 80/PG (%) and oat oil (%) in (a) and (b) cases. It can be observed the minimized effect of response in low levels of Tween 80/PG and high levels of oil phase, from lighter green through darker green-like zones. Points of maximum and minimum are better distinguished on the wireframe representation.

According to the second situation, in cases (c) and (d), the response is represented as a function of Tween 80/PG (%) and lecithin (%). The surface plot in gradient color in variation from blue through darker green, shows the implication of X_1_ factor in the work of adhesion modulation, but without a significant effect on the X_2_ term. The embossed appearance of the wireframe model shows maximum points related with high W values, when Tween 80/PG (%) was set at 40%, and medium or minimum points corresponding with the use of lower levels of the stabilizer.

In the final representations, denoted (e) and (f), the Y_3_ response was determined in relationship with the X_2_ and X_3_ factors. On the surface plot, it can be seen that better results in the matter of adhesion were obtained with high levels of oil phase and low levels of lecithin, being marked by the lighter green tones. The results were well defined on the wireframe plot. The decrease in oil concentration and the increase in lecithin level assured intermediate and high responses for Y_3_.

**Figure 24:**
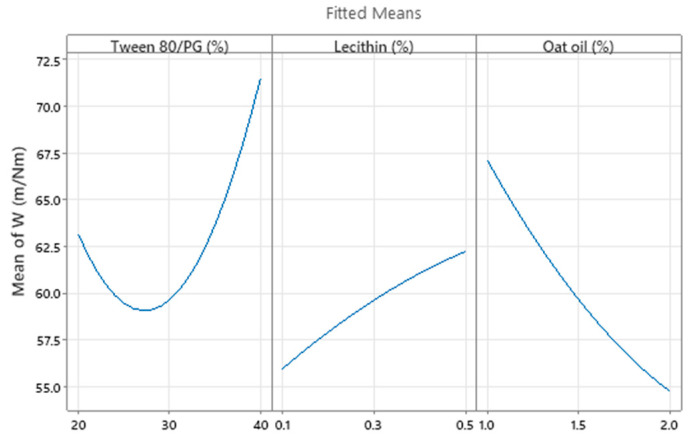
Main effects plot for W response (Y_3_), considering individual effect of each independent variable.

According to the results revealed in the graphical analysis, the optimal systems proposed are characterized by the work of adhesion placed in a domain of around 60 mN/m. In this way, the systems can exhibit good adhesion properties, which can be obtained with low or even high levels of surfactants and by maximizing the level of oil phase. The optimization study of the W response (Y_3_), included as in the previous cases, and the main effects of variables are represented using factorial plotting. Thus, Figure 24 presents the main effects of the analyzed factors on the Y_3_ response which are related with our previous findings. It can be observed that the X_1_ and X_3_ terms had a critical influence on the response: firstly, X_1_ can, in a first phase, induce a decrease in the work of adhesion over a variation from low to medium levels (from 20% to 30%) and continue with a considerable increase in response (at 40%); secondly, the oil phase had an opposite behavior explained by the minimization of adhesion in high concentrations of 2%. Separately, lecithin had a diminished effect on adhesion in the selected domain of concentration and it was proportional to the X_1_ effect in the limited domain of 30–40%, but not as powerful.

Predictive response optimization was applied with the aim to define formulations that can exhibit appropriate adhesion qualities, related with a Y_3_ value of 60 mN/m, and then compare them with experimental versions. In this case, optimization parameters were configured in the sense of minimizing Y_3_, and resulted in two cases presented in Table 13. In the first case, constraints were imposed only on the oil variable as maximized. X_1_ and X_3_ variables had no constraints. The fitted solution proposed the following composition: X_1_: 33.53%, X_2_: 0.5%, X_3_: 2%, with a composite desirability of 1.0000. A second run was also deemed to be valuable. Constraints were imposed on both the X_1_ and X_3_ variables in order to be maximized. The solution was based on the following formula: X_1_: 40%, X_2_: 0.107%, X_3_: 2%, with a composite desirability of 1.0000.

Thereby, based on the two solutions obtained, it can be considered that MELSA 12 and MELSA 8 were characterized by good adhesion properties, which can sustain a proper spreadability without a fast flow effect or a rapid evaporation on the tissue, maintaining in the same manner a prolonged contact with the surface in order to promote hydration and therapeutic activity.

**Figure 25:**
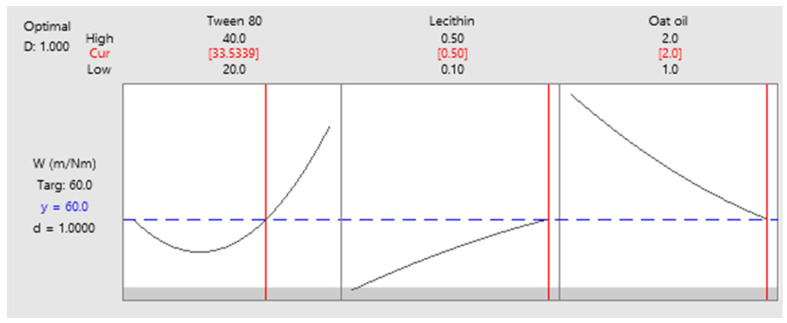
Predicted optimization plots for W response (Y_3_) as function of three setting parameters: X_1_—without constraints, X_2_—without constraints, and X_3_—maximized, with a composite desirability of 1.0000.

The MELSA 12 system can be considered a prototype proposed for the first predictive result. It had a medium level of Tween 80/PG closer to 33.53%, and 0.5% lecithin, exhibiting a work of adhesion of 59.79 mN/m. By applying the exclusion criterion based on the clarity grade, the system can be considered as optimal only from the perspective of a proper work of adhesion. The interpretation for the predictive optimization process was described in Figure 25. The Y_3_ response is marked by a blue intermittent line in all three cases. A red perpendicular line marks the optimized level of the variable. At their intersection, a gray line passes in the sense of response minimization as was previously pointed out.

**Figure 26:**
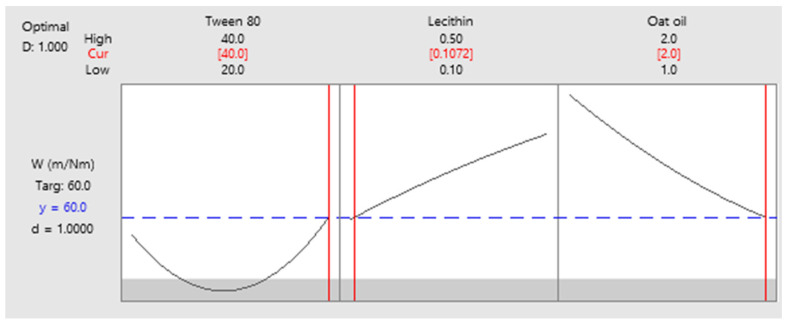
Predicted optimization plots for W response (Y_3_) as function of three setting parameters: X_1_—maximized, X_2_—without constraints, and X_3_—maximized, with a composite desirability of 1.0000.

The MELSA 8 microemulsion represents a model system with characteristics that were well correlated with the response of the second solution of predictive analysis. Composed of a maximum level of Tween 80/PG, a maximum level of oil and 0.3% lecithin, the system exhibited a work of adhesion of 62.13 mN/m. The interpretation of predictive response is graphically described in Figure 26, and can be explained by reference to the anterior rules, as well as in the case presented above.

## 4. Discussion

The main objectives of this study were to formulate and evaluate from a physical point of view a series of O/W microemulsions with salicylic acid as pharmaceutical remedies for acne therapy purposes. The systems were projected by applying a quality by design approach and proposing a Box–Behnken modeling based on a three-factor, three-level factorial plan. Thus, 13 microemulsions with salicylic acid were prepared using a stabilizer hydrophilic mixture of Tween 80 as surfactant and propylene glycol as cosurfactant on a domain of 20–40%. In addition, lecithin 0.1–0.5% was considered as a second surfactant with lipophilic properties. Subsequently, oat oil of 1–2% was chosen as a lipophilic phase at a minimum level. The dispersion medium consisted of a hyaluronate aqueous solution, which could sustain a moisturizing effect.

The solubilization effect of the stabilizer mixture and oil phase for salicylic acid was considered interesting to be studied in further experiments by selecting a larger concentration domain of oil phase. By reference to the study of Sinha P. et al., salicylic acid 1% was solubilized in lemongrass oil and then combined with a mixture of Tween 80 and Labrasol in order to obtain nanoemulsified systems for acne therapy purposes [19]. A shortcoming of their study which can also be considered in the present case, was supposed to be the lack of implementing the standard method based on the pseudoternary phase diagram design, which can offer a general and useful perspective upon the stable areas of microemulsions and differentiation between unstable dispersions. On the other hand, the method has some drawbacks, including higher costs and a prolonged period of screenings. Nevertheless, a challenging attempt in the formulation process of nanodispersions can be sustained in the same manner by implementing quality by design concepts and proposing a model that can offer a critical interpretation on formulation factors and their implication in the development of reliable microemulsions. In addition, an HLB criterion was applied to sustain the formation of stable systems, as was previously mentioned.

The obtained microemulsions were characterized by adequate physical parameters. One month after the preparation process, clear and turbid microemulsions were observed and appropriately graded. The qualitative parameter was considered important on the optimization process and quoted as an exclusion criterion for turbid systems. Turbidity was correlated with an increase in droplet size up to 37 nm, being specific for systems with a low S/CoS level and medium or high concentrations of oil phase. In the study of Fernández-Peña L. et al., the clarity of micro dispersions with thymol increased with the molar ratio between alkyl polyglucoside and soybean lecithin in favor of the first surfactant. It is worth mentioning that oleic acid 3% was selected as the oil phase, and was stabilized by a total amount of surfactants up to 30% [73].

Proceeding to the assessment of the physical attributes of the systems, the pH was placed between 3.49 and 3.71 and was found proper for anti-acne formulations. Normal skin pH varies between 4.1–5.8, depending on physiological status of the body. The resemblance of skin with an acidic mantle is correlated with this parameter. Thus, topical products with pH over 6 are not recommended for patients with sensitive skin and acne predisposition because negative outcomes may occur, including skin hydration imbalances, alterations in skin surface pH, and bacterial proliferation [93]. It has been proved that skin acidification with dermatologic products which contain proton donors APIs, promote a regulation in skin pH balance [94].

The conductivity and refractive index were assessed as two important physical and quality parameters that confirmed the O/W type of the systems and their isotropy. Their variation was significantly associated with the modification of Tween 80/PG concentration.

Rheological evaluation of microemulsions, along with the estimation of the hydrodynamic diameter of particles and an extensive superficial analysis oriented through quantification of adhesion properties, involved special attention by implementing a response surface methodology to follow and outline optimal systems as was reported in a couple of studies oriented to topical formulations design [67,85].

A desired topical microemulsion must accomplish a series of requirements considering viscosity, droplet size, and work of adhesion. A high viscosity will sustain a proper spreadability on tissue, offering in the same manner a pleasant effect at the application site than a fast-flowing formulation with inadequate displaying. Microemulsions are known to possess limitations related with their fluid-like structure. The use of proper excipients, specifically calculated ratios of surfactants and a design of gel-based systems can sustain the application of microemulsions on skin and a prolonged retention, as well [58].

The small droplets of oat oil containing salicylic acid, dispersed in the hyaluronate aqueous vehicle can be concentrated at skin level in nanometric size. The increased surface area promoted by the stabilizer mixture of Tween 80/PG/lecithin, is considered to assure stabilization of particles due to lower superficial tensions and a close contact with the tissue. As a consequence, a penetration effect is expected to occur at stratum corneum level.

Nonetheless, a moderate work of adhesion can sustain fine adhesion and wettability phenomena which ensure the spreading and an adequate localization of the formulation on skin surface. It can be appreciated, that all three studied critical quality attributes are interconnected and will finally define the pharmaceutical relevance of microemulsions from a physical point of view.

Rheological measurements revealed that viscosity varied between 16.94–292.69 cP. Flow behavior of microemulsions was considered specific for Newtonian fluids. Values of viscosity of 22.4–26.69 cP were reported in rheological study of O/W microemulsions with minoxidil, being associated with the composition based on a total amount of S/CoS mixture of 53% [83]. As the concentration of S/CoS blend increases between 54–60%, viscosity can attain 637.47–808.20 cP, as it was observed in the evaluation process of O/W microemulsions with palm oil 6%, without changing the model of viscosity [95]. In-depth studies were conducted to assess rheological properties of O/W microemulsions using a mixture of Tween 80/PG (2:1) (10–30%) as a powerful solubilizer for cinnamon essential oil 2–5%. In this case, viscosity parameter depended on the oil phase amount (20–30%) and the addition of the essential oil, and varied between 166.2–969.5 cP [47].

Examining Tween 80/PG as the main parameter implied in variation of viscosity, the systems were characterized as fast-flow fluids at low and medium levels of the stabilizer (16.94–51.23 cP), or viscous fluids at the maximum level of 40%. In the last case, the small group of viscosity of systems formulated with the maximum amount of Tween 80/PG (188.17–292.69 cP) drawn attention and guided to the discovery of the optimal formulation based on Tween 80/PG: 40%, lecithin 0.1%, oat oil 2%, with a predicted viscosity of 263.83 cP.

Between MELSA 2, MELSA 4, MELSA 6 and MELSA 8 systems, the most eloquent systems that were closely related with the predicted system were MELSA 6 (287.77 cP) and MELSA 8 (217.73 cP). MELSA 8 system had a priority to be selected due to the maximum oil content used in dispersion process.

To complete the quality profile of microemulsions, new characteristics were gathered over DLS analysis. There were defined microemulsions with particles under 100 nm. The presence of a minimum quantity of oil of 1–2%, selected for microemulsion preparation, represented a reason for the fine dispersion of particles under the effect of the stabilizer mixture. The Ds values were placed in microemulsion domain. Comparable results were obtained in the study of Szumała P., where for some experimental microemulsions with monoacylglycerols, PG in a hydro-alcoholic dispersion medium, Ds were found to belong to a narrowed domain of 5–30 nm. The integration of cosurfactants like ethanol or PG was considered critical for phase behavior and droplet size [96]. In a recent study reported by Vlaia L. et al. centered on the development of O/W microemulsions with fluconazole 2% and essential oils 3–5%, reduced levels of isopropyl myristate 3–5% were stabilized with sucrose esters 45%. DLS analysis revealed Ds values of 9.9–19.2 nm [97]. In other case, like nanoemulsion development, particle domain tends to transcend the limit of 100 nm, but their formation remains to be influenced in the same manner by oil concentration and surfactant proportion [19].

As a result, it was found that mean hydrodynamic diameter of 1.58–37.72 nm was influenced by oat oil and Tween 80/PG (%) levels. Low levels of Tween 80/PG (%), associated with high levels of oil phase, determined formation of particles of 11.35–37.72 nm. In a single case for MELSA 5, the presence of oat oil 1% determined a decrease in diameter at 3.43 nm, obtaining in the same time a high clarity grade. An increase in stabilizer level, determined a reduction in diameter in domain of 4.11–6.45 nm, excepting MELSA 12 (26.02 nm) characterized by a maximum level of lecithin and oil phase. On the other side, a close attention was attributed for the group formulated with the maximum level of Tween 80/PG which determined a significant decrease in Ds, between 1.58–5.88 nm.

In a conclusive way, it can be stated that Tween 80/PG (%) and oat oil (%) actioned as two opposed factors in the generation of O/W microemulsions. During optimization, a predictive plan sustained the possibility to obtain a microemulsion characterized by the following formula: X_1_: 35%, X_2_: 0.2214%, X_3_: 2%, with a Ds of 5 nm. From the experimental results, two systems were in immediate area of response, specifically MELSA 8 and MELSA 11. It was admitted that only one system with the maximum level of X_1_ and X_3_ factors was declared as optimal, with respect to desired quality parameters, namely MELSA 8, with a Ds of 2.17 nm.

Supplementary, PDI and zeta potential offered preliminary information concerning the internal structure of microemulsions. PDI variation in domain 0.039–0.557, reflected the presence of both monodisperse and polydisperse systems as it was previously reported [61,97]. There was not found any relationship between PDI variation and main ingredients, but it was suggested that Tween 80/PG level may contribute in dispersion process, influencing stabilization and formation of homogeneous systems characterized by low PDI values under 0.119, or systems with a complex distribution of particles and higher PDI values up to 0.557. Recent studies in the field of nanoemulsified systems conducted by Jarzębski M. et al., outlined the significance of natural surfactants on particle behavior and stabilization process. It was pointed out that surfactant level will be implied in a decrease of interfacial tension, determining droplet size reduction and modulating elasticity of droplets [98]. In extensive studies performed on nanoemulsions with hemp seed oil 1–5%, it was emphasized the possibility to obtain large values of PDI near 1, correlated with a scattering of large droplets dispersed in the systems. In the case of nanoemulsions with an aqueous continuous phase, an increase in oil phase level may elevate PDI, while an increase in stabilizer level determined PDI lowering [99]. In the case of microemulsions as colloidal systems with dispersed particles under 100 nm, Vlaia L. et al. depicted as well PDI values between 0.129–0.363 nm attributed for homogeneous systems with monomodal distribution—for PDI values of 0.129 and 0.171, or bimodal distribution—for PDI up to 0.363. In addition, zeta potential was found to be negative and close to zero value due to the presence of non-ionic surfactant and low levels of lecithin. It was found that microemulsion stabilization in this case will not be characterized by powerful electrostatic repulsions. Steric repulsions or dispersion forces are implied in stabilization [61,97]. We concluded that in-depth studies are further mandatory to discover internal structure of microemulsions and assess stability data. Here it can be included the implementation of microscopic techniques, reproducibility analysis, the study of dispersion time and the manner in which time may affect droplet size distribution. Not least, performing of extensive stability studies with a follow-up of the parameters in time was considered desirable.

The study of superficial properties by applying goniometric principles has a double relevance in colloids science. The internal structure of the systems and their stability can be evaluated by the measurement of γ_L/G_. Subsequently, the study of contact angle—CA at the contact with planar surfaces constitutes a method to evaluate the wettability of a system, particularly hydrophilicity or hydrophobicity nature, as it was reported in the study of Popa L. et al. [85]. Goniometry was rarely applied in the research of microemulsions, and it was considered that their behavior at the contact with surfaces is not fully unveiled [100]. Although, Butt U. et al. studied some topical microemulsions for ophthalmic delivery which were characterized as hydrophilic systems after the evaluation of contact angle. The values of contact angle varied between 12.2–25.2° at the contact with a hydrophilic surface, and 25.9–43.8° at the contact with a hydrophobic surface, which were chosen as models to explain the wetting behavior of microemulsions at corneal level [61]. Similarly, in topical delivery, skin is considered a low hydrophobic surface [101], and for this reason the analysis of drops was performed on glass slide. It was considered that contact angle rely on three properties: the surface tension of liquid (γ_L/G_), surface free energy and interaction forces at liquid-solid surface [61].

γ_L/G_ and CA parameters, were considered two key factors in the assessment of work of adhesion which was considered relevant for the optimization of microemulsions. In pendant drop model, γ_L/G_ varied between 32.57–37.36 mN/m, describing stability of the systems characterized by low superficial tensions, and consequently low interfacial tensions between oil and water phases. In the study of Yati K. et al., the values of γ_L/G_ reported after applying De Nouy ring method for microemulsions stabilized with Tween 80 and sorbitol, were placed between 39.76–43.4 mN/m [95]. Likewise, in CA model γ_L/G_ was evaluated as a parameter implied in drop dynamics, succeeding adhesion promotion. It was observed that Tween 80/PG (%) had a powerful effect in the angle generation. Microemulsions had a hydrophilic character, with angles under 90°, placed between 40.74–57.91° and supported the wetting of the planar surface.

Adhesion phenomenon was truly described by the presence of mechanical forces that can action in the sense of decreasing γ_L/G_ and enhance interactions with the surface. During the response surface analysis, it was revealed in this case the impact of all three formulation factors (X_1_, X_2_, X_3_) in modulation of adhesion, where oat oil exerted an important action on wettability behavior, in two opposite directions.

Adhesion values varied between 57.2–81.00 mN/m. Tween 80/PG (%) and lecithin (%) were factors that supported the elevation of work of adhesion, when selected at maximum levels. Oat oil (%) was on the far side and reduced the mechanical activity, if it was selected at maximum level. Because systems with low adhesion were mainly defined by low levels of stabilizers, a priority in this case was to optimize and predict a system with low work of adhesion of 60 mN/m, characterized by a high content of Tween 80/PG and oat oil, as well. On this way, these levels could be satisfactory considering spreadability and wettability, by reference to both viscosity and Ds parameters.

As a result of the optimization process, two predicted solutions were proposed to meet the conditions and were characterized by the following formulae: X_1_: 33.53%, X_2_: 0.5%, X_3_: 2%, and X_1_: 40%, X_2_: 0.107%, X_3_: 2%. For these solutions, two formulations from the experimental domain were proposed. MELSA 12 complied with the first run, but MELSA 8 was the most reliable model, due to the fact that MELSA 12 did not accomplish the requirements in the matter of clarity and viscosity. It can be appreciated that a higher level of oil, not only has the role of lipophilic carrier for an API, but also determines an affinity for the surface, specifically an intimate contact with lipidic structures of the skin membrane.

Finally, it was valued that the study of adhesion emphasized the connection between physical attributes related to superficial phenomena and the usefulness in practice was related to the application of microemulsions on tissues.

## 5. Conclusions

The present research defined the development and optimization of some topical microemulsions with salicylic acid for acne alleviation. Microemulsions were designed proposing a Box–Behnken modeling based on a three-factor, three-level factorial plan. The systems were analyzed from a physical point of view. A particular interest was accorded to the attributes that define topical administration of a pharmaceutical product at skin level.

Following this direction, three formulation factors were coded: X_1_: Tween 80/PG (%), X_2_: Lecithin (%), and X_3_: Oat oil (%), as independent variables. Viscosity (Y_1_), mean droplet size (Y_2_) and work of adhesion (Y_3_) were analyzed as dependent parameters influenced by X_1_, X_2_ and X_3_, by applying a response surface methodology.

Using a rheological analysis, it was confirmed that the X_1_ factor highly influenced viscosity parameter. In high concentrations of 40%, viscous fluids with Newtonian behavior were formed, being defined by an adequate spreadability on surfaces which sustain their topical application. Based on the value of predicted viscosity of 263.83 cP, two systems were selected as optimal microemulsions, specifically MELSA 6 (287.77 cP) and MELSA 8 (217.73 cP). MELSA 8 was considered to be a model system due to the presence of 2% oil phase, as was proposed in the predictive analysis step.

Mean droplet size was affected by X_1_ and X_3_ factors, in an opposite manner. Thus, increasing concentration of the X_1_ factor through to the maximum level determined a decrease in droplet size, while the maximum level of X_3_ was a priority for particle growing. The possibility to obtain a reduced droplet size by keeping X_1_ and X_3_ at the maximum level was studied. Throughout the optimization process, MELSA 8 and MELSA 11 were found to comply with a predicted response of droplet size of 5 nm, keeping the oil phase level at 2%. MELSA 8 had the highest level of Tween 80/PG of 40%, while MELSA 11 contained an intermediate level of the stabilizer. If the clarity parameter is considered, MELSA 8 remained the first choice among the group of microemulsions.

In the case of superficial analysis, the work of adhesion was a critical parameter that sustained the adhesion and wettability behavior of microemulsions. It was found that all three factors X_1_, X_2_ and X_3_ modulated the response in a different manner. X_3_ was the key factor that supported adhesion at maximum level of the oil phase, increasing the contact with the surface. In this case, the maximum level of oil phase at 2% was maintained over the predictive analysis. Two responses were generated, emphasizing two microemulsions with Tween 80/PG 33.53% and 40% that can sustain wettability and adhesion with a work of adhesion of 60 mN/m. As a result, MELSA 12 with a medium level of Tween 80/PG was considered to be one of the optimal systems, with a response of 59.79 mN/m, but without respecting clarity criterion. By contrast, MELSA 8 exerted a work of adhesion of 62.13 mN/m, being related to the second predicted response.

To our appreciation, MELSA 8 was the final system closest to the optimal formulation discovered using a quality by design approach. It was based on Tween 80/PG 40%, lecithin 0.3%, oat oil 2%, salicylic acid 0.5%, hyaluronic acid 1%, and water 56.2%. It was defined by a viscosity of 217.73 cP, a mean droplet size of 2.17 nm, and a work of adhesion of 62.13 mN/m, being considered an interesting prototype for topical drug delivery and a model to follow in further studies.

## Figures and Tables

**Table 1:** Composition of fluid O/W microemulsions formulated with salicylic acid.

Formulation	^1^ Tween 80/^2^ PG (2:1) (X_1_)	^1^ Lecithin (X_2_)	^1^ Oil (X_3_)	^1,3^ HA	^1,4^ SA	^1^ Water
MELSA 1	20	0.1	1.5	1	0.5	76.9
MELSA 2	40	0.1	1.5	1	0.5	56.9
MELSA 3	20	0.5	1.5	1	0.5	76.5
MELSA 4	40	0.5	1.5	1	0.5	56.5
MELSA 5	20	0.3	1	1	0.5	77.2
MELSA 6	40	0.3	1	1	0.5	57.2
MELSA 7	20	0.3	2	1	0.5	76.2
MELSA 8	40	0.3	2	1	0.5	56.2
MELSA 9	30	0.1	1	1	0.5	67.4
MELSA 10	30	0.5	1	1	0.5	67
MELSA 11	30	0.1	2	1	0.5	66.4
MELSA 12	30	0.5	2	1	0.5	66
MELSA 13	30	0.3	1.5	1	0.5	66.7

^1^ The values presented in the table for each component are expressed as percentage (%), and calculated for 20 mL of microemulsion; ^2^ PG—propylene glycol; ^3^ HA—Hyaluronic acid; ^4^ SA—Salicylic acid.

**Table 2:** Factorial plan for microemulsions with three independent variables and three levels of variation, coded for each factor; and dependent variables for study of the optimization process.

Factor	Variable	Low (−1)	Medium (0)	High (+1)
X_1_	Tween 80/PG (%)	20	30	40
X_2_	Lecithin (%)	0.1	0.3	0.5
X_3_	Oil (%)	1	1.5	2

**Table 4:** Results of pH, conductivity, refractive index, viscosity, and particle characteristics determined at 25 ± 0.5 °C, which describe the physical character of MELSA 1–MELSA 13 microemulsions.

Code	pH	Conductivity (μS/cm)	Refractive Index	Viscosity (cP)	Droplet Size (nm)	PDI	Zeta Potential (mV)
1	3.50 ± 0.01	1042.7 ± 1.2	1.3610 ± 0.0001	20.65 ± 2.05	11.35 ± 1.05	0.268 ± 0.011	−1.98 ± 0.02
2	3.71 ± 0.01	506.0 ± 3.0	1.3874 ± 0.0001	292.69 ± 4.18	3.32 ± 1.85	0.091 ± 0.010	−2.78 ± 0.05
3	3.49 ± 0.01	1088.0 ± 1.7	1.3624 ± 0.0001	27.89 ± 2.63	21.88 ± 1.25	0.039 ± 0.017	−3.78 ± 0.05
4	3.70 ± 0.01	621.3 ± 0.6	1.3860 ± 0.0001	188.17 ± 3.03	5.88 ± 1.16	0.070 ± 0.010	−2.14 ± 0.05
5	3.49 ± 0.01	1065.0 ± 1.0	1.3610 ± 0.0001	24.33 ± 1.15	3.43 ± 1.73	0.297 ± 0.075	−3.83 ± 0.01
6	3.63 ± 0.02	524.3 ± 0.6	1.3863 ± 0.0001	287.77 ± 4.30	1.58 ± 0.20	0.119 ± 0.023	−3.16 ± 0.06
7	3.48 ± 0.01	986.3 ± 1.5	1.3617 ± 0.0000	16.94 ± 2.16	37.72 ± 4.55	0.250 ± 0.025	−2.80 ± 0.02
8	3.71 ± 0.01	542.3 ± 0.6	1.3880 ± 0.0001	217.73 ± 3.54	2.17 ± 1.54	0.352 ± 0.027	−1.72 ± 0.03
9	3.54 ± 0.01	772.0 ± 1.0	1.3728 ± 0.0001	48.47 ± 3.06	5.14 ± 2.15	0.134 ± 0.018	−1.43 ± 0.03
10	3.54 ± 0.01	728.7 ± 1.5	1.3729 ± 0.0001	34.82 ± 1.05	5.30 ± 2.53	0.218 ± 0.037	−2.33 ± 0.05
11	3.53 ± 0.01	717.3 ± 1.5	1.3764 ± 0.0001	51.23 ± 3.33	6.45 ± 2.55	0.202 ± 0.011	−2.38 ± 0.03
12	3.59 ± 0.01	786.0 ± 0.0	1.3735 ± 0.0001	39.29 ± 2.08	26.02 ± 3.78	0.557 ± 0.038	−1.38 ± 0.05
13	3.56 ± 0.01	661.3 ± 1.5	1.3768 ± 0.0001	45.84 ± 2.67	4.11 ± 1.65	0.240 ± 0.021	−2.38 ± 0.02

**Table 5:** Superficial parameters of microemulsions, explored during goniometric analysis at 25 ± 0.5 °C.

Code	Models Applied in Goniometric Study					
	Pendant Drop		Contact Angle Model			
	Vol (μL)	γ_LG_ (mN/m)	Vol (μL)	γ_LG_ (mN/m)	CA (°)	W (mN/m)
1	6.56 ± 0.04	34.80 ± 0.35	5.30 ± 0.17	35.78 ± 1.96	45.51 ± 3.06	60.71 ± 1.96
2	6.53 ± 0.10	32.57 ± 0.34	6.53 ± 1.27	41.23 ± 1.89	50.27 ± 0.76	67.52 ± 2.99
3	6.65 ± 0.02	35.55 ± 0.03	5.00 ± 0.48	36.78 ± 0.72	46.59 ± 1.44	62.04 ± 0.53
4	6.56 ± 0.05	33.36 ± 0.16	6.17 ± 0.30	45.59 ± 1.50	49.01 ± 2.40	76.91 ± 2.90
5	7.15 ± 0.02	37.36 ± 0.16	6.95 ± 0.13	41.31 ± 1.67	40.74 ± 0.70	72.61 ± 3.08
6	6.47 ± 0.12	33.69 ± 0.24	6.58 ± 0.43	52.90 ± 1.82	57.91 ± 0.38	81.00 ± 2.49
7	6.84 ± 0.07	36.25 ± 0.21	6.94 ± 0.35	33.93 ± 0.70	43.27 ± 1.29	58.64 ± 1.25
8	6.72 ± 0.17	33.73 ± 0.24	6.49 ± 0.14	40.14 ± 0.86	56.78 ± 2.30	62.13 ± 1.66
9	6.36 ± 0.09	33.61 ± 0.10	5.79 ± 0.15	37.63 ± 1.81	51.93 ± 1.62	60.80 ± 2.38
10	7.10 ± 0.21	35.85 ± 0.29	6.23 ± 0.67	42.87 ± 3.95	54.04 ± 2.61	68.14 ± 7.88
11	6.76 ± 0.18	35.37 ± 0.85	6.94 ± 0.21	33.38 ± 0.46	54.59 ± 1.86	52.72 ± 1.44
12	7.05 ± 0.11	36.12 ± 0.35	7.11 ± 0.70	35.98 ± 0.99	48.50 ± 2.49	59.79 ± 1.39
13	6.52 ± 0.08	34.11 ± 0.08	7.70 ± 0.44	35.42 ± 0.29	46.75 ± 4.14	59.64 ± 1.62

**Table 6:** Results of ANOVA-Single factor test, emphasizing the variance of γ_LG_ in the two groups: γ_LG_ determined in pendant drop model, and γ_LG_ determined in contact angle model study at 25 ± 0.5 °C.

SUMMARY						
Groups	Count	Sum	Average	Variance		
Column 1	13	452.37	34.798	1.9753		
Column 2	13	512.94	39.457	29.74		
**ANOVA**						
**Source of Variation**	**Sum of Squares**	**df**	**Mean of Squares**	**F**	***p*-Value**	**F Crit**
Between Groups	141.10	1	141.10	8.8975	0.0065	4.2596
Within Groups	380.61	24	15.86			
Total	521.72	25				

**Table 7:** Optimization parameters presented as function of independent variables analyzed using response surface methodology for microemulsions MELSA 1–MELSA 13.

Variable	Independent Variables				Dependent Variables		
	X_1_	X_2_	X_3_		Y_1_	Y_2_	Y_3_
Formulation	Tween 80/PG (%)	Lecithin (%)	Oil (%)	^1^ Clarity	Viscosity (cP)	Droplet Size (nm)	Adhesion Work (mN/m)
MELSA 1	20	0.1	1.5	++	20.65 ± 2.05	11.35 ± 1.05	60.71 ± 1.96
MELSA 2	40	0.1	1.5	+++	292.69 ± 4.18	3.32 ± 1.85	67.52 ± 2.99
MELSA 3	20	0.5	1.5	++	27.89 ± 2.63	21.88 ± 1.25	62.04 ± 0.53
MELSA 4	40	0.5	1.5	+++	188.17 ± 3.03	5.88 ± 1.16	76.91 ± 2.90
MELSA 5	20	0.3	1	+++	24.33 ± 1.15	3.43 ± 1.73	72.61 ± 3.08
MELSA 6	40	0.3	1	+++	287.77 ± 4.30	1.58 ± 0.20	81.00 ± 2.49
MELSA 7	20	0.3	2	+	16.94 ± 2.16	37.72 ± 4.55	58.64 ± 1.25
MELSA 8	40	0.3	2	+++	217.73 ± 3.54	2.17 ± 1.54	62.13 ± 1.66
MELSA 9	30	0.1	1	+++	48.47 ± 3.06	5.14 ± 2.15	60.80 ± 2.38
MELSA 10	30	0.5	1	+++	34.82 ± 1.06	5.30 ± 2.53	68.14 ± 7.88
MELSA 11	30	0.1	2	+	51.23 ± 3.33	6.45 ± 2.55	52.72 ± 1.44
MELSA 12	30	0.5	2	+	39.29 ± 2.08	26.02 ± 3.78	59.79 ± 1.39
MELSA 13	30	0.3	1.5	+++	45.84 ± 2.67	4.11 ± 1.65	59.64 ± 1.62

^1^ Notation of clarity parameters: “+” was attributed for an opalescent system, “++”—for a low clarity system, and “+++”—for a high clarity system.

**Table 8:** Analysis of variance for viscosity response.

Source	DF	Sum of Squares	Mean of Squares	F-Value	*p*-Value
Model	9	132,377	14,709	26.49	0.010
Linear	3	102,978	34,326	61.83	0.003
X_1_	1	100,475	100,475	180.97	0.001
X_2_	1	1887	1887	3.40	0.162
X_3_	1	616	616	1.11	0.370
Square	3	25,294	8431	15.19	0.026
$X_{1\;}^{2}$	1	18,463	18,463	33.25	0.010
$X_{2\;}^{2}$	1	26	26	0.05	0.843
$X_{3\;}^{2}$	1	2	2	0.00	0.954
2-Way Interaction	3	4105	1368	2.46	0.239
X_1_X_2_	1	3123	3123	5.62	0.098
X_1_X_3_	1	981	981	1.77	0.276
X_2_X_3_	1	1	1	0.0	0.973
Error	3	1666	555		
Total	12	134,042			

**Table 9:** Response optimization for viscosity by maximizing the response and keeping X_3_ at maximum level.

Solution	X_1_	X_2_	X_3_	Y_1_	95% Confidence Interval	Composite Desirability
	Tween 80/PG (%)	Lecithin (%)	Oat Oil (%)	Viscosity (cP)		
1	40	0.1	2	263.83	(170.1, 357.6)	0.8953

**Table 10:** Analysis of variance for mean droplet size (Ds) response.

Source	DF	Sum of Squares	Mean of Squares	F-Value	*p*-Value
Model	9	1448.02	160.89	9.95	0.042
Linear	3	1011.19	337.06	20.85	0.016
X_1_	1	471.71	471.71	29.18	0.012
X_2_	1	134.64	134.64	8.33	0.063
X_3_	1	404.84	404.84	25.04	0.015
Square	3	42.83	14.28	0.88	0.539
$X_{1\;}^{2}$	1	27.96	27.96	1.73	0.280
$X_{2\;}^{2}$	1	20.57	20.57	1.27	0.341
$X_{3\;}^{2}$	1	29.91	29.91	1.85	0.267
2-Way Interaction	3	393.99	131.33	8.12	0.060
X_1_X_2_	1	15.88	15.88	0.98	0.395
X_1_X_3_	1	283.92	283.92	17.56	0.025
X_2_X_3_	1	94.19	94.19	5.83	0.095
Error	3	48.50	16.17		
Total	12	134,042			

**Table 11:** Response optimization for mean droplet size (Ds) by minimizing the response and keeping X_3_ at maximum level.

Solution	X_1_	X_2_	X_3_	Y_2_	95% Upper Confidence interval	Composite Desirability
	Tween 80/PG (%)	Lecithin (%)	Oat Oil (%)	Ds (nm)		
1	35.0	0.2214	2	5	(11.84)	1.0000

**Table 12:** Analysis of variance for the work of adhesion (W) response.

Source	DF	Sum of Squares	Mean of Squares	F-Value	*p*-Value
Model	6	702.080	117.013	10.08	0.006
Linear	3	523.165	174.388	15.03	0.003
X_1_	1	140.784	140.784	12.3	0.013
X_2_	1	78.940	78.940	6.80	0.040
X_3_	1	303.442	303.442	26.15	0.002
Square	3	178.915	59.638	5.14	0.043
$X_{1\;}^{2}$	1	135.300	135.300	11.66	0.014
$X_{2\;}^{2}$	1	0.663	0.663	0.06	0.819
$X_{3\;}^{2}$	1	3.636	3.636	0.31	0.596
Error	6	69.630	11.605		
Total	12	771.710			

**Table 13:** Response optimization for work of adhesion (W), with two possible solutions, by minimizing the response to 60 mN/m.

Solution	X_1_	X_2_	X_3_	Y_3_	95% Confidence Interval	Composite Desirability
	Tween 80/PG (%)	Lecithin (%)	Oat Oil (%)	W (mN/m)		
1	33.53	0.5	2	60.00	(48.66, 71.34)	1.0000
2	40	0.107	2	60.00	(44.63, 75.37)	1.0000

## Data Availability

Data are available from the authors upon request.
